# Traditional Chinese Medicine targeting the TGF-β/Smad signaling pathway as a potential therapeutic strategy for renal fibrosis

**DOI:** 10.3389/fphar.2025.1513329

**Published:** 2025-05-20

**Authors:** Hao Jiao, Meijuan Zhang, Lili Chen, Zhirui Zhang

**Affiliations:** ^1^ Department of Pharmacy, The First Affiliated Hospital of Wannan Medical College (Yijishan Hospital of Wannan Medical College), Wuhu, Anhui, China; ^2^ Department of Pharmacy, Wannan Medical College, Wuhu, Anhui, China; ^3^ Department of Research Ward, Beijing Friendship Hospital, Capital Medical University, Beijing, China; ^4^ Guangdong Key Laboratory for Biomedical Measurements and Ultrasound Imaging, National-Regional Key Technology Engineering Laboratory for Medical Ultrasound, School of Biomedical Engineering, Shenzhen University Medical School, Shenzhen, China

**Keywords:** Traditional Chinese Medicine, TGF-β/Smad signaling pathway, renal fibrosis, therapeutic strategy, Chinese herbal compounds

## Abstract

Renal fibrosis (RF) is an inevitable outcome of nearly all progressive chronic kidney diseases (CKD). However, effective therapies that can halt or reverse the development of RF and CKD progression remain limited. Traditional Chinese Medicine (TCM) offers a unique therapeutic approach, demonstrating significant anti-fibrotic potential through its antioxidant and anti-inflammatory pharmacological properties. However, comprehensive reviews focusing on the role of TCM in targeting signaling pathways associated with RF are still scarce. In this review, the literature was screened according to the clarity of the relevance of TCM, including the types and mechanisms of TCM. We summarize the pivotal role of the TGF-β/Smad signaling pathway in RF and provide an overview of single Chinese botanical drug, their active ingredients, and TCM compounds that ameliorate RF by modulating this pathway, aiming to establish a solid foundation for future basic and clinical research in the field of RF. While TCM holds unique advantages in treating RF, its limitations need to be addressed through scientific research and technological innovation. Future studies will focus on elucidating mechanisms, improving quality control, validating clinical efficacy, and fostering international collaboration to promote the scientific and global application of TCM in modern medicine.

## 1 Introduction

Chronic kidney disease (CKD) is a pathology that cause abnormal kidney function or altered kidney structure for more than 3 months, and results in a gradual and permanent loss of kidney function over time (Rayego-Mateos and Valdivielso, 2020; Chou et al., 2024). The global prevalence of kidney disease is on the rise, currently ranking as the seventh most significant contributor to mortality worldwide. Shifting demographic patterns and escalating risk factors are driving a steady rise in the global impact of kidney disease, projected to emerge as the fifth leading cause of mortality among non-communicable diseases by 2040 (Kovesdy, 2022; Francis et al., 2024). Among Chinese adults participating in this cross-sectional research, CKD was observed in 8.2% of the population, however, there are still approximately 82 million adults in mainland China suffering from CKD (Wang L. et al., 2023). Nonetheless, effective therapies that can halt or reverse the development of renal fibrosis (RF) and CKD progression remain limited.

RF is an inevitable outcome in nearly all patients with progressive CKD (Duffield, 2014; Yin et al., 2025). RF is the primary cause of declining kidney function and a common progression of CKD to end-stage renal disease (ESRD), characterized by glomerulosclerosis and tubulointerstitial fibrosis (Huang et al., 2023). It is a chronic pathophysiological change characterized by abnormal deposition of extracellular matrix (ECM). Under the influence of various pathogenic factors including inflammation and infection, blood circulation disorders, and immune response, the inherent cells of the kidney are destroyed, resulting in a large amount of deposition and accumulation of connective tissue, leading to gradual hardening of the kidney parenchyma until the kidney completely loses its function activity (Klinkhammer and Boor, 2023). Inflammation and oxidative stress are the main factors driving the progression and occurrence of RF (Su et al., 2019; Panizo et al., 2021), and the severity of fibrosis is closely associated with a decline in the glomerular filtration rate (GFR). The urgency to address RF lies in its irreversible nature and its role as a major contributor to the global burden of kidney disease.

However, because the pathogenesis of RF remains unclear, western medicine does not have an effective means of treatment; therefore, symptomatic treatment is the primary approach. The pathogenesis of RF is complex and diverse and is associated with multiple mechanisms and various signaling pathways (Humphreys, 2018). Traditional Chinese medicine (TCM) not only has the characteristics of multi-component, multi-target and multi-pathway treatment, but also has the advantages of obvious curative effects and few side effects (Wang J. et al., 2018, Wang P. et al., 2018; He et al., 2022); therefore, it is widely used in the treatment of RF.

The appearance and development of RF are related to the induction of various stimuli, such as toxins, cytokines, and growth factors, as well as the overactivation of key signaling pathways (Byun et al., 2025). Pathways that promote RF include: the transforming growth factorβ (TGF-β) pathway, renin-angiotensin system (RAS), Wnt pathway, Notch, and nuclear factor-κB (NF-κB) (Niculae et al., 2023; Zhao et al., 2025). The TGF-β/Smad signaling pathway plays a crucial role in RF, serving as one of the core molecular mechanisms driving the fibrotic process (Hu et al., 2018; Isaka, 2018; Li et al., 2025). As a key regulatory factor in fibrosis, TGF-β1 activates the Smad protein family, modulates the expression of downstream genes, and promotes the excessive deposition of ECM, leading to the scarring and functional loss of renal tissue (Liang et al., 2024). In the early stages of RF, the release of TGF-β1 is triggered by various pathological factors, such as inflammation, oxidative stress, and hemodynamic changes. The TGF-β/Smad signaling pathway further exacerbates fibrosis by suppressing the expression of matrix metalloproteinases (MMPs) and enhancing the production of tissue inhibitors of metalloproteinases (TIMPs), thereby disrupting the balance of ECM degradation (Chen et al., 2025). In summary, the critical importance of the TGF-β/Smad signaling pathway in RF lies not only in its ability to regulate ECM metabolism but also in its interactions with other fibrosis-related pathways, such as Wnt/β-catenin and MAPK, collectively driving disease progression (Lee and Massague, 2022).

Currently, most reviews have focused on highlighting the signaling pathways of TCM in RF, yet few have specifically addressed the importance of TCM in targeting specific signaling pathways involved in RF. Given the critical role of TGF-β/Smad signaling in promoting RF, this review explores the anti-RF effects of TCM by targeting the TGF-β/Smad signaling pathway to delay the progression of CKD and identify more effective clinical agents for RF. TCM is characterized by its multi-component and multi-target properties, and this review will analyze how TCM can more effectively regulate the TGF-β/Smad pathway through synergistic interactions. Additionally, we will compare the effects of TCM on TGF-β/Smad signaling in different cell types, such as renal tubular epithelial cells and fibroblasts, to reveal its cell-specific mechanisms. Beyond single botanical drugs, this review will also focus on TCM compounds and their active components, systematically evaluating the efficacy and safety of TCM in treating RF. Furthermore, we will explore the potential of TCM in personalized treatment approaches for RF, providing a foundation for its clinical application.

## 2 Dysregulation of the ECM in renal fibrogenesis

**FIGURE 1:**
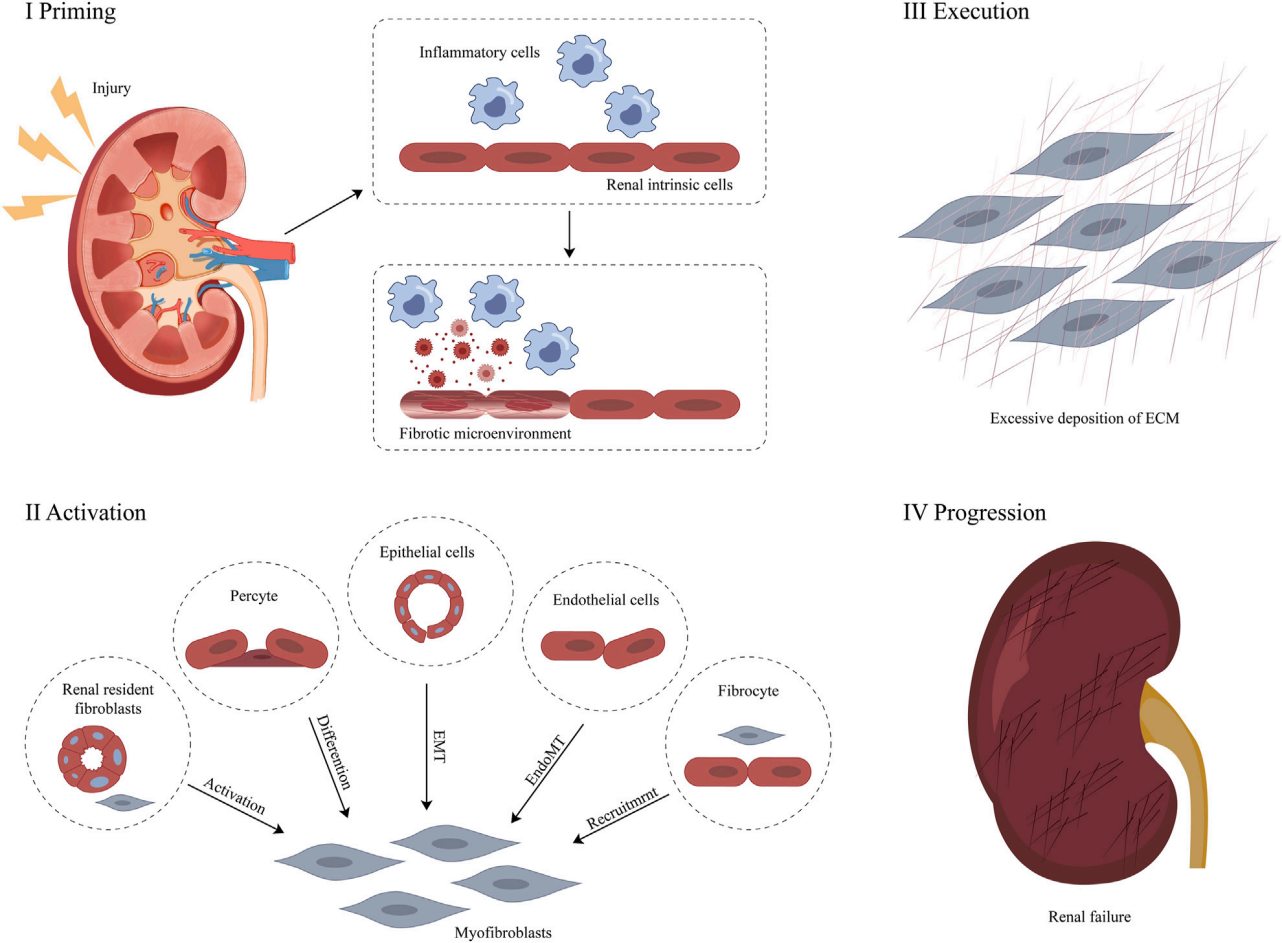
Pathological mechanism of RF (Zhou et al., 2022).

RF occurs in the renal interstitium and glomeruli, which manifests itself as renal interstitial fibrosis and glomerulosclerosis, respectively. Overall, glomerular mesangial cell hyperplasia and ECM are significantly enlarged. Over time, the renal tubules and interstitial capillaries are lost and the ECM accumulates excessively (Bohle et al., 1979; Li et al., 2019). The ECM is part of the tissue surrounding the cell and is an important factor in the regulation of cellular behaviour. ECM components not only provide dynamic tissue integrity but also participate in and drive many biological responses as signaling molecules. Their dysregulation is a direct or indirect cause of most chronic diseases, including RF (Theocharis et al., 2019; Luangmonkong et al., 2023). Renal cells producing ECM include interstitial fibroblasts, glomerular mesangial cells, and tubular epithelial cells. Stimulated by oxygen free radicals, cytokines, endothelin, angiotensin, and other factors, these cells undergo activation or trans-differentiation, and their cell phenotype changes, all become myofibroblasts (MFB) expressing α-smooth muscle actin (α-SMA) (Sun et al., 2016). MFB produce ECM in large amounts, including collagen, glycoproteins, and proteoglycans, and secrete matrix metalloproteinase inhibitors, including plasminogen activator inhibitors (PAI-1), and tissue matrix metalloproteinase inhibitors (TMP), reducing the degradation activity of MMP and the production of ECM is greater than the degradation, resulting in RF (Flevaris and Vaughan, 2017; Toba and Lindsey, 2019). The abnormal accumulation of the ECM is the molecular basis of RF due to tubulointerstitial injury (Kim et al., 2022) (Figure 1).

Apart from cellular factors, there are diverse mechanisms leading to the abnormal accumulation of the ECM (Antar et al., 2023). In terms of cytokines and growth factors, TGF-β binds to its receptor and activates the Smad signaling pathway, promoting the synthesis of ECM and inhibiting its degradation. Platelet-derived growth factor (PDGF) stimulates the proliferation, migration, and ECM synthesis of fibroblasts and other cells, and it can also act synergistically with TGF-β. Angiotensin II (Ang II) not only directly prompts the resident renal cells to synthesize ECM, but also activates the TGF-β/Smad pathway (Isshiki et al., 2024). Moreover, it causes vasoconstriction in the kidneys, leading to ischemia and hypoxia, which exacerbates RF. The inflammatory response should not be underestimated either. Inflammatory cells infiltrate the kidneys and release inflammatory mediators and reactive oxygen species, which directly stimulate the resident renal cells to synthesize ECM (Jang et al., 2020). They can also activate profibrotic cytokines such as TGF-β, indirectly promoting ECM deposition. At the same time, oxidative stress damage is caused, disrupting the metabolic balance between renal cells and ECM (Li et al., 2023).

Abnormal signaling pathways are equally crucial. The TGF-β/Smad signaling pathway can be indirectly activated by various factors, promoting the synthesis and deposition of ECM (Bai et al., 2023). Subfamilies of the mitogen-activated protein kinase (MAPK) signaling pathway, such as extracellular signal-regulated kinase (ERK), c-Jun N-terminal kinase (JNK), and p38 MAPK, after being activated by multiple stimulating factors, regulate cell proliferation, differentiation, apoptosis, and ECM synthesis (Wu M. et al., 2023). In addition, abnormal lipid metabolism can lead to lipid deposition in the kidneys, activate the inflammatory response and oxidative stress, and thus promote the synthesis of ECM and the development of RF (Chen et al., 2022). These mechanisms are intertwined and influence each other, collectively resulting in the abnormal accumulation of ECM and the disruption of the structure and function of the renal tissue during the process of RF.

## 3 Role of the TGF-β/Smad signaling pathway in renal fibrosis

### 3.1 Role of TGF-β in renal fibrosis

**FIGURE 2:**
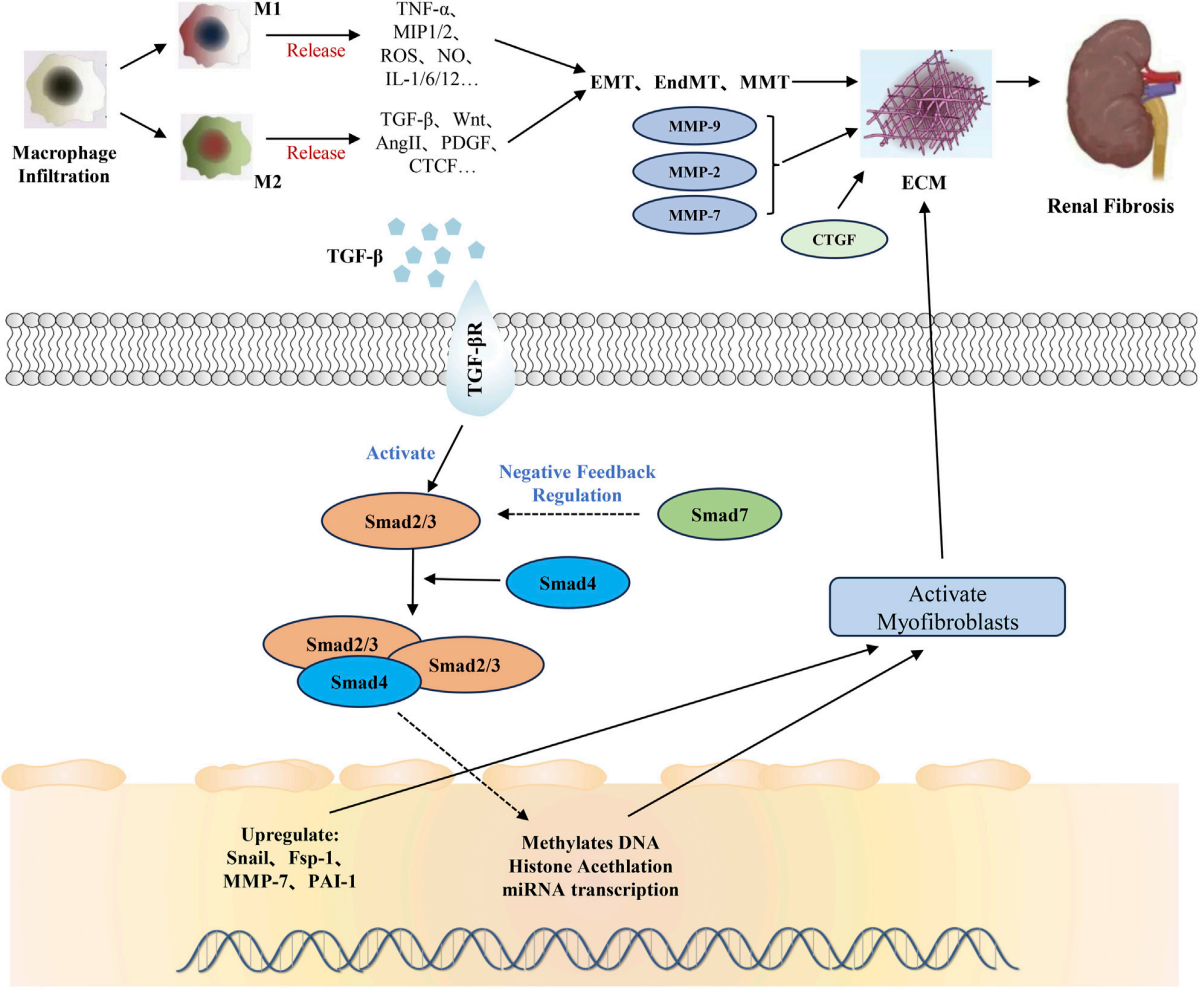
Regulatory functions of the TGF-β/Smad signaling pathway in the renal fibrosis. ECM deposition is the most important pathological change of renal fibrosis. TGF-β and extracellular matrix are the main factors affecting ECM. TGF-β can indirectly lead to ECM by inducing epigenetic modification and miRNA expression directly in the synthetic fiber matrix through the TGF-β/Smad signaling pathway, or by inducing the accumulation of myofibroblasts by inducing cell phenotypic transformation. The abnormal expression of metalloproteinases and CTGF in extracellular matrix is also an important reason for aggravating RF.

TGF-β, a multifunctional cytokine regulating various cell activities, is crucial in diseases like bone diseases, fibrosis and cancer. In mammals, it has three subtypes (TGF-β1, TGF-β2, and TGF-β3), with TGF-β1 being most associated with RF and releasable by all renal cells including invading inflammatory cells (Abbad et al., 2025; Sheikh et al., 2025). TGF-β overexpression can directly induce ECM production or act on resident renal cells, accelerating mesangial cell proliferation and inducing the loss of renal tubular epithelial cells and podocytes, thus leading to RF (Hong et al., 2025). Moreover, it accelerates fibrosis by inhibiting ECM degradation and causing excessive collagen deposition. Elevated TGF-β levels respond to kidney damage from diabetes, hypertension, obstruction, ischemia, repetitive tubular injury and urinary tract obstruction by influencing pathological renal ECM synthesis and fibrosis processes (Hong et al., 2025). Also, TGF-β induces the transformation of human fibroblasts into myofibroblasts by upregulating α-SMA expression and stimulates fibroblasts to transcribe collagen I and fibronectin (Figure 2) (di Miceli et al., 2024). Hence, targeting the inhibition of TGF-β overexpression is a viable strategy for preventing and treating RF.

TGF-β activates both Smad and non-Smad (MAPK, PI3K/AKT) pathways (Giarratana et al., 2024; Garg et al., 2025). In the MAPK pathway, TGF-β context-dependently activates ERK (via Ras-Raf-MEK), JNK (through upstream kinases like MEKKs), and p38 MAPK (by a kinase cascade), influencing cell proliferation, apoptosis, inflammation, and ECM-related processes in fibrosis (Chen H. et al., 2024). In the PI3K/AKT pathway, TGF-β activates it in some cell types, promoting fibroblast survival and ECM production in fibrosis and interacting with the Smad pathway (Zhao M. et al., 2022, Zhao Y. et al., 2022). TGF-β's effect on non-Smad pathways adds complexity to its signaling and impacts cell behavior in physiological and fibrotic contexts.

TGF-β is a crucial target for treating fibrotic diseases in both experimental and clinical settings (Hong et al., 2025). In experimental settings, gene manipulation techniques like knockout or knockdown, antibody-based therapies with neutralizing antibodies, and small molecule inhibitors targeting the TGF-β signaling pathway are used to study its role and test potential treatments, although complete TGF-β blockade may have side effects (Harding et al., 2021; Guo et al., 2024; Yu et al., 2025). In clinical settings, monoclonal antibodies against TGF-β are being trialed but face challenges due to potential increased infection risks and other adverse events, repurposed drugs with indirect effects on the pathway are used off-label with less conclusive evidence, and combination therapies are being explored to address disease complexity while considering drug interactions and toxicities (Trachtman et al., 2011; Lan et al., 2022). Key differences and challenges include the inability of experimental models to fully replicate human disease complexity, safety concerns in patients when blocking TGF-β, and the high heterogeneity among patients compared to the more homogeneous experimental models, highlighting the need for further research to optimize TGF-β-targeted therapeutic strategies.

### 3.2 Role of Smad protein in renal fibrosis

In the TGF-β signaling pathway, Smad protein is a direct substrate for TGF-β. Smad proteins enter the nucleus after being stimulated by TGF-β and undergo repeated dephosphorylation-phosphorylation cycles, shuttling back and forth from the nucleus to the cytoplasm. Most transcription responses of TGF-β are mediated by Smad2/3, with Smad6 and Smad7 serving as “inhibitory” Smads, which can be considered anti-fibrotic factors (Ma and Meng, 2019). Smad2/3 play pro-fibrotic roles as they are activated by TGF-β binding to its receptors, with phosphorylated Smad2/3 forming complexes with Smad4 and translocating to the nucleus to upregulate genes encoding ECM components like collagens and fibronectin, induce EMT in epithelial cells which contributes to the accumulation of myofibroblast-like cells, and downregulate MMPs to inhibit ECM degradation, all of which promote fibrosis (Yu et al., 2022; Chen C. et al., 2023). During the development of RF, Smad2/3 is highly activated in humans and laboratory animals with CKD (Chen L. et al., 2018). Smad3 is a key mediator of TGF-β signaling and plays a pathogenic role in renal inflammation and fibrosis. In contrast, Smad6/7 act as anti-fibrotic factors; Smad7 binds to activated TβRI to prevent Smad2/3 phosphorylation and complex formation, blocking TGF-β's pro-fibrotic downstream effects, while Smad6 can interfere with Smad complex formation and inhibit both TGF-β and BMP signaling involved in fibrosis (Lin et al., 2025). Additionally, Smad7 enhances MMP expression for ECM degradation and inhibits myofibroblast activation and proliferation, thus counteracting the fibrotic process and maintaining tissue homeostasis (Hu et al., 2018).

### 3.3 microRNAs (miRNAs) and long coding RNAs (IncRNAs) are regulated by TGF-β/Smad signaling pathway in renal fibrosis

In recent years, miRNAs and lncRNAs have attracted a great deal of attention because of their potential biological functions in various pathophysiological processes in the body, such as fibrosis of multiple organs. Studies have shown that miRNAs and IncRNAs could affect the occurrence and development of RF through the TGF-β/Smad signaling pathway. Inhibition of miR-130a-3p protects against RF through the TGF-β/Smad pathway (Ai et al., 2020). Upregulation of miR-21 enhanced TGF-β1-induced EMT and aggravates RF progression by directly downregulating Smad7 phosphorylation and indirectly up-regulating Smad3 phosphorylation (Wang et al., 2014). Furthermore, miR-140-5p directly targets TGF-β1 to mediate RF (Liao et al., 2020). We also found that Erbb4-IR, a novel Smad3-associated lncRNA that is highly upregulated in renal interstitial fibrosis or diabetic nephropathy with progressive RF, mediates RF these conditions by targeting renal Smad7 and miRNA-29b (Feng et al., 2018). Meanwhile, lnc-TSI, a lncRNA expressed in human renal tubular epithelial cells, blocked the activation of TGF-β/Smad3 pathway by inhibiting the phosphorylation of Smad3, thus inhibiting the appearance and development of RF (Wang J. et al., 2018). In conclusion, lncRNAs and miRNAs are abnormally expressed in RF-related pathways and play crucial roles in the occurrence and development of RF. Given that the TGF-β/Smad signaling pathway regulates several miRNAs and that IncRNAs play a vital role in RF, targeting the regulation of these RNAs for the prevention and treatment of RF would be a feasible and promising therapeutic strategy.

## 4 Research status of Traditional Chinese Medicine (TCM) targeting TGF-β/Smad signaling pathway for the prevention and treatment of renal fibrosis

### 4.1 Understanding renal fibrosis in TCM

TCM functions on the concept that the imbalance of yin and yang is the fundamental contradiction of disease; therefore, the basic principle of curing the root cause is to adjust yin and yang, solve contradictions that cannot be solved by the yin and yang of the human body, and restore the harmony and balance of yin and yang in the body (Zhong et al., 2015). Most practitioners of TCM have adopted “renewing vital energy and nourishing the blood,” “purifying heat and removing dirt,” and “harmonizing the body’s Yin and Yang” as the principles in the treatment of CKD (Liu X. Y. et al., 2022). Practitioner experience has shown that these principles ameliorate symptoms, increase diuresis, decrease proteinuria, and maintain renal function (Li and Wang, 2005; Shen et al., 2024). TCM believes that RF is caused mainly by insufficient congenital endowment, acquired uncontrolled diet, fatigue disorder, or long-term illness. If left untreated or mistreatment that leads to deficiency of qi and blood yin and yang in human organs, especially spleen and kidney deficiency, dampness and turbidity, turbidity and evil congestion, and imbalance of qi and machine, can result in accumulation of pathological products such as water dampness, damp heat, phlegm turbidity, turbidity, and blood stasis (Zhong et al., 2015; Wang Y. et al., 2022).

Therefore, clinical therapies for RF are based on the principle of strengthening the right and removing evil, and the treatment approach mainly involves nourishing the spleen and kidney, removing heat and humidity, dissolving turbidity and detoxification, promoting blood circulation and eliminating blood stasis (Wei et al., 2002; Chen D. Q. et al., 2018). TCM is characterized by multi-component and multi-target action, many of which improve RF by targeting and regulating the TGF-β/Smad signaling pathway (Xu et al., 2021; Yao et al., 2023). Therefore, a complete understanding of the aetiology, pathogenesis, treatment, and prescription of RF in TCM and elucidating its mechanism of action through pharmacological research will help popularise TCM in the prevention and cure of RF worldwide.

### 4.2 Single Chinese botanical drugs and active components that target the TGF-β/Smad signaling pathway for the prevention and cure of RF

#### 4.2.1 Tonifying formula TCM

In TCM, *Astragalus membranaceus* (Fu et al., 2014) has the effects of qi (i.e., vital energy or life power) replenishment, promote diuresis, and alleviate edema, and has been widely used in the therapy of various kidney diseases (Liu H. L. et al., 2024) (Table 1). Constituent glycosides and polysaccharide have obvious protective effects on the kidneys and can delay the progression of RF (Chen C. et al., 2023; Chen Z. et al., 2023; Liu M. et al., 2024; Shi et al., 2024). For example, astragaloside IV (40 mg/kg) attenuates RF in rats by inhibiting excessive mesangial proliferation and RF via the TGF-β1/Smad/miR-192 signaling pathway, while doses exceeding 80 mg/kg resulted in slight vacuolar degeneration in renal tubular epithelial cells (Mao et al., 2019). The results of a multi-center, assessor-blind, randomized controlled trial clinical study showed that adjuvant treatment with *Astragalus membranaceus* can further stabilize renal function (Chan et al., 2024). *Epimedium alpinum* L. (Chen X. L. et al., 2024) had an effect on tonifying kidney yang, strengthening muscles and bones, and dispelling rheumatism, and is rich in a variety of effective ingredients against RF (Chen et al., 2011). Icariin, the main prenylflavonoid of *Epimedii Folium*, is a well-known TCM used for its traditional benefits in treating kidney diseases, osteoporosis and rheumatism. Treatment with Icariin prevents CKD-related RF due to its anti-fibrotic and anti-inflammatory properties (Chen et al., 2019; Seyedi et al., 2023). While inhibiting the deposition of collagen and the expressions of profibrotic factors and fibrotic markers, icariin (20 mg/kg) significantly suppresses the increase of Samd2/3 protein in the kidneys of mice and reduces the expression of E-cadherin. Most of these drugs are qi tonifying and aphrodisiac, with a sweet taste, active mostly in the lungs, spleen, liver and kidney. The pharmacological studies of these drugs have fully confirmed the authenticity and reliability of the “Fuzheng” TCM theory in RF therapy. Furthermore, most of these drugs have antioxidant and free radical scavenging effects, which are consistent with the purpose of achieving anti-RF activity by inhibiting the oxidative stress mechanism of RF (Liu Y. et al., 2024; Patera et al., 2024).

**TABLE 1:** A single Chinese medicine and its active ingredients treat renal fibrosis through TGF-β/Smad signaling pathway.

Classification	Drug	Drug information	Model	Dosage	Mechanism	References
Tonifying formula TCM	Astragaloside IV	Major active constituent of *Astragalus membranaceus*	A DN model was established using streptozotocin administration in rats	40 mg/kg	Modulating of the TGF-β/Smad/miR-192 signaling pathway	Mao et al. (2019)
	*Coreopsis tinctoria* Nutt	A plant of the genus Asteraceae in the family of Compositae	A diabetic rat model	150, 300, 600 mg/kg	Inhibiting inflammatory and fibrotic processes, suppresses the TGF-β/Smad signaling pathway	Yao et al. (2015)
	*Astragalus* polysaccharides	Derived from a leguminous plant, *Astragalus membranaceus*	A type 2 diabetic rat model	25, 50, 100 mg/kg	Affecting the TGF-β/Smad signaling pathway	Meng et al. (2020)
	*Astragalus mongholicus* Bunge	*Astragalus membranaceus*	Type 2 diabetic mice	0.03 mL/10 g.d	Rebalancing TGF-β/Smad signaling	Nie et al. (2014)
Heat-clearing TCM	Mudan granules	Composed of *Astragalus membranaceus*, *Corydalis yanhusuo*, *Panax notoginseng*, *Paeoniae Radix Rubra*, *Salvia miltiorrhiza Bunge*, *Ligusticum chuanxiong Hort.*, *Carthamus tinctorius*, *Haematoxylum campechianum*, *Spatholobus suberectus Dunn*	Streptozotocin-induced diabetes rats	1.08, 2.16, 4.32 g/kg	Inhibiting TGF-β/Smad pathway and the production of ECM	Qi et al. (2024)
	Alpha-mangostin	Xanthone derivatives of *Garcinia mangostana L.*	UUO mouse model	10, 20 mg/kg	Targeting the TGF-β1/ERK/Smad-mediated EMT signaling pathway	Juan et al. (2023)
	Baicalin	The dry roots of the Chinese herb *Scutellaria baicalensis Georgi*	Rat kidney interstitial fibroblast (NRK-49F) cells	40 µM	Inhibiting of the TGF-β1 signaling pathway	Hu et al. (2017)
	Baicalin	The dry roots of the Chinese herb *Scutellaria baicalensis Georgi*	UUO RIF model	10, 20 and 40 mg/kg	Inhibiting the TGF-β/Smad signaling pathway	Wang H. et al. (2022)
	Wogonin	Major components of Scutellaria baicalensis Georgi	TGF-β1-treated tubular epithelial cells	4 μg/μl	Inhibiting the TGF-β/Smad3 signaling pathway	Meng et al. (2016)
	*Glycyrrhiza uralensis* root extract	Root extracts of *Glycyrrhiza uralensis* (*Radix glycyrrhizae*)	HK-2 renal proximal tubular epithelial cell line and a type-II-diabetes model with Apoe^em1/Narl^/Narl mice	100, 150, 200 μg/kg	Targeting TGF-β/Smad/Stat3 pathway	Lin et al. (2022)
	Amygdalin	Found in the seeds of Rosaceae plants, such as peach, apricot, and plum	Streptozotocin-induced diabetic rats	1, 3, 10 mg/kg/d	Suppressing TGF-β/Smad signaling pathway and regulating the key enzymes of ECM degradation	Chen J. et al. (2021)
	*Cordyceps cicadae* polysaccharides	A class of polysaccharide substances extracted from *Cordyceps cicadae*	SD rats fed with a high-fat diet	75, 150 and 300 mg/kg	Blocking the TLR4/NF-κB and TGF-β/Smad signaling pathway	Yang J. et al. (2020), Yang K. et al. (2020)
	Acetylshikonin	The main ingredient of *Lithospermum erythrorhizon*	A DN model	100 mg/kg/d	Inhibiting TGF-β/Smad signaling pathway	Li S. S. et al. (2018), Li Z. et al. (2018)
	Cordyceps sinensis	Composed of the parasitic fungus Cordyceps sinensis	5/6 subtotal nephrectomy	2.0 g/kg/d	Inhibition of TGF-β/Smad signal pathway	Pan et al. (2013)
	Isoliquiritigenin	A chemical component extracted from *Glycyrrhiza uralensis Fisch*	Human mesangial cells	1, 10 and 20 μM	Blocking the TGF-beta1-Smad signaling	Li et al. (2010)
	Oxymatrine	An herbal product derived from the root of *Sophora flavescens Aiton*	Rat renal tubular epithelial cells (NRK52Es)	0.50 mg/mL	Upregulating SnoN expression and inhibiting TGF-β/Smad signaling pathway activation	Liu et al. (2016)
	Salidroside	A compound extracted from plants of the genus *Rhodiola rosea L.*	Streptozotocin (STZ) combined with right nephrectomy	50, 100 and 200 mg/kg	Inhibiting the TGF-β/Smad pathway	Leng et al. (2019)
Dispel dampness TCM	Levistolide A	A natural compound extracted from *Angelica sinensis* (Oliv.) Diels	TGF-β1-stimulated HK-2 cells and 5/6 nephrectomy (Nx) mice	0.5, 1.0 and 2.0 mg/kg	Inhibiting RAS, TGF-β/Smad, and MAPK pathways to downregulate ECM deposition	Aobulikasimu et al. (2023)
	Poricoic acid ZA	A small compound isolated from *Wolfiporia cocos*	HK-2 cells and podocytes induced by TGF-β1 and angiotensin II (ANGII)	10 µM	Inhibiting RAS and further suppresses TGF-β/Smad pathway through inhibiting Smad2/3 phosphorylation via blocking Smad2/3-TGFβRI protein interaction	Wang et al. (2017)
	Sang-Bai-Pi extract	The root bark of *Morus alba* L.	UUO mouse model	200 and 300 mg/kg	Targeting the TGF-β/Smad by blocking the interaction of Smad3 with TGF-βRII and TGF-βRI and Wnt/β-catenin pathways.	Wei et al. (2025)
	Multi-glycoside of *Tripterygium wilfordii* Hook. f	A stable glycoside extracted from *Tripterygium wilfordii* Hook. f.	Adriamycin (ADR)-induced nephropathy (ADRN) model by right nephrectomy	50 mg/kg	It could reduce the ECM deposition and improve GS by way of intervening TGF-beta1/Smad signaling pathway	Wan et al. (2011)
	Multi-glycoside of *Tripterygium wilfordii* Hook. f	A stable glycoside extracted from *Tripterygium wilfordii* Hook. f.	adriamycin (ADR)-induced nephropathy (ADRN)	50, 100 mg/kg	Bidirectional regulation of TGF-β/Smad signaling activity	Wan et al. (2014)
	Celastrol	A bioactive compound extracted from *Tripterygium wilfordii* Hook. f.	UUO mouse model	1 mg/kg	Upregulation of CB2R expression through inhibiting Smad3 signaling pathway activation	Tang et al. (2018)
Laxative TCM	Emodin	A natural component extracted from Chinese herbs, such as *Cassia obtusifolia* and *Rheum palmatum*	5/6 renal mass reduction	0.3 and 1.0 mg/kg	Down-regulating expressions of TGF-β1 and Smurf 2 and up-regulating Smad7 expression	Ma et al. (2018)
	Rhubarb Extracts	Extracts of *Rheum palmatum* L.	CKD rats	PE extract (800 mg/kg), EA extract (200 mg/kg), and BU extract (600 mg/kg)	Rebalancing the disorder of TGF-β/Smad signaling and metabolic dysfunction	Zhang et al. (2018)
	Chrysophanol	Derived from the roots and rhizomes of *Rheum palmatum* L.	UUO mouse model	10, 20 and 40 mg/kg/d	Inhibiting the TGF-β/Smad signaling pathway	Dou et al. (2020)
Blood-activating TCM	Hirudin	An extract from salivary glands of medicinal leeches (*Hirudo medicinalis*)	UUO rat model	10, 20, or 40 IU/kg/d	Inhibiting of TGF-β/Smad and NF-κB signaling	Yang J. et al. (2020)
	Norcantharidin	A synthetic demethylated analogue of cantharidin	UUO mouse model	0.075 mg/kg/d	Modulation of TGF-β/Smad signal cascade, inhibition of protein serine/threonine phosphatases (PPP) as well as NF-κB	Zeng et al. (2020)
	Danshen injection	The dried root of the plant *Salvia miltiorrhiza* Bunge	STZ-induced diabetic rats	0.5 or 1 mL/kg/d	Suppressing the oxidative stress, inflammatory responses and fibrosis progression	Xu et al. (2016)
	Curcumin	An active component of *Curcuma longa* L.	UUO rat model	100 mg/kg	Intervene several sites of the TGF-beta/Smads signal transduction pathway	Li et al. (2011)
	Curcumin	An active component of *Curcuma longa* L.	UUO rat model	50, 100 mg/kg	Downregulated the TGF-β/Smad signaling pathway and inhibited Dragon and fibrogenic molecules in both rats and HK-2 cells	Chen F. et al. (2021)
Others	Saponins from *Panax japonicus*	A class of phytochemicals present in various plant species, including Panax japonicus (*P. japonicus*)	Sprague-Dawley (SD) aging rats were given saponins from *Panax japonicus* (SPJs)	10, 60 mg/kg/d	Inhibiting TGF-β/Smad, NF-κB signaling pathways and activating Nrf2-ARE signaling pathways	Gao et al. (2021)
	Honokiol	An effective ingredient extracted from *Houpoea officinalis*	UUO mouse model	5 mg/kg	SIRT3-dependent regulation ofmitochondrial dynamics and the NF-κB/TGF-β/Smad signaling pathway	Quan et al. (2020)
	Carnosic Acid	It mainly exists in plants such as *Rosmarinus officinalis* L. and *Salvia officinalis* L.	normal kidney epithelial (NKE) cells	10 mg/kg	Inhibiting Oxidative Stress, Promoting Nrf2/HO-1 Signalling and Impairing TGF-β/Smad/Collagen IV Signalling	Das et al. (2019)
	Ginsenoside-Rg1	An agent isolated from *Panax ginseng*	UUO rat model	50 mg/kg	Targeting Klotho/TGF-β/Smad signaling	Li et al. (2018)
	*Taxus chinensis*	A valuable plant, which belongs to the Taxaceae family.	Rat DN model	0.32, 0.64, and 1.28 g/kg	Suppressing TGF-β/Smad signaling pathway	Weng et al. (2018)

#### 4.2.2 Heat-clearing TCM for heat removal

*Scutellaria baicalensis* Georgi, also known as skullcap, is a significant herbal remedy in TCM (Zhao et al., 2019). It is effective in removing heat and dampness, purging fire, detoxifying, stopping bleeding, and stabilizing the fetus (Bao et al., 2022). Baicalin was also found to restrict the TGF-β/Smad signaling pathway to prevent renal interstitial fibrosis in mice (Hu et al., 2017; Ganguly et al., 2022; Wang H. et al., 2022). Baicalein (20–80 µM) exerts an antifibrotic effect by inhibiting cell proliferation, the deposition of extracellular matrix, the synthesis of collagen, the expression of endogenous TGF-β1, and the phosphorylation of Smad3. Heat-clearing drugs are an important embodiment of their anti-infective pharmacological effects. The accumulation of RF heat toxins in the body leads to a long-term state of micro-inflammation, especially after various infections caused by the invasion of external evils (Yeh et al., 2024). TGF-β1, as an important anti-inflammatory cytokine, which can inhibit NF-κB-mediated kidney inflammation by inducing Smad7-dependent IκBα expression (Lan, 2011). Moreover, most heat-clearing drugs have anti-pathogen, antitoxin, antipyretic, anti-inflammatory, and immunity-enhancing effects (Lu et al., 2020), therefore, their clinical application has a clear effect on improving the micro-inflammatory state of RF and fever caused by infection.

#### 4.2.3 TCM dispel dampness

Dampness drugs are classified into water-diluting, dampness-resolving, rheumatic and aromatic dampness drugs. Diuretic drugs, a type of water-diluting drugs, mainly work by opening water passages and expelling dampness, with diuresis as the main effect.

In TCM, RF edema’s etiology and pathogenesis involve lung regulation, spleen transmission, renal opening/closing and abnormal bladder gasification, leading to water distribution imbalance, skin flooding, edema, oliguria, and potentially pleural effusion and ascites in severe cases (Tang et al., 2021; Song L. et al., 2024). Modern medicine holds that kidney disease edema can cause thrombosis, embolism, acute renal failure, heart failure and even death. Using diuretics to reduce the body load from edema in modern medicine shares the same essence with Traditional Chinese medicine, showing their interconnection and complementarity (Siddall and Radhakrishnan, 2012; Meena and Bagga, 2020). TCM dampness-dispelling drugs have anti-inflammatory, analgesic effects and can inhibit the immune system, yet most are toxic like cytotoxic immunosuppressants. Immune complex deposition worsens RF, and modern medicine has proven the effects of rheumatic drugs on the immune system.

*Smilax glabra* Roxb (Nie et al., 2020) and Alisma (Feng et al., 2021) are commonly used in clinical practice to reduce water and swelling, and studies had found that their extracts are widely used for diuresis and kidney protection (Dou, Miao et al., 2018; Liang et al., 2021; Lu et al., 2024). *Smilax glabra* Roxb has been traditionally utilized in alternative medicine to treat various conditions including brucellosis, syphilis, acute and chronic nephritis (Xia et al., 2010). Poricoic acid ZA inhibits the RF process by blocking the protein interaction between Smad2/3 and TGFβRI, suppressing the phosphorylation of Smad2/3, thereby inhibiting RAS and further suppressing the TGF-β/Smad signaling pathway (Wang et al., 2017). The EA3 fraction of *Morus alba L.* root bark, known as Sang-Bai-Pi in TCM, has been reported to alleviate RF by targeting the TGF-β/Smad and Wnt/β-catenin signaling pathways through blocking the interaction of Smad3 with TGF-βRII and TGF-βRI (Wei et al., 2025). *Tripterygium wilfordii Hook. F.* is a rheumatic drug with anti-inflammatory, anti-tumor, and immunosuppressive effects and is widely used to treat autoimmune diseases, allergic diseases, and kidney diseases (Nong et al., 2019; Jin et al., 2021; Zhang Q. et al., 2021). *Tripterygium* glycosides of 50 and 100 mg/kg were administrated intragastric for 6 weeks to relieve glomerulosclerosis in rats with Adriamycin-induced nephropathy by controlling the levels of TGF-β1, Smad3, p-Smad2/3 and Smad7 (Wan et al., 2014). Celastrol is a triterpenoid compound with multiple biological activities extracted from *Tripterygium wilfordii Hook. F*. It upregulates the expression of the antifibrotic factor cannabinoid receptor 2 (CB2R) by inhibiting activation of Smad3 signaling, thus reducing RF (Tang et al., 2018).

#### 4.2.4 Laxative TCM

The pharmacological effects of purgative TCM mainly include purgation, diuresis, anti-inflammation, antibacterial activity, and anti-tumor effects, etc. Their diuretic effect in improving renal function is similar to that achieved by using diuretics and dampness-resolving drugs. Moreover, purgative TCM also have improved anti-inflammatory and antibacterial effects on renal micro-inflammation and renal damage aggravated by various infections (Yue et al., 2023).

*Rheum officinale*, with the Chinese name as Da Huang, has functions like purging accumulation, clearing heat, cooling blood, detoxifying and removing stasis in TCM (Wang Y. et al., 2023). Its main active components are emodin and rhein, and its extract and isolated compounds can inhibit RF (Wang et al., 2022b; Wang et al., 2022c; Zhang et al., 2023). *Rheum officinale* extract suppressed adenine-induced RF in rats by reducing the expression of TGF-β1, TβRI, TβRII, Smad2/p-Smad2, Smad3/p-Smad3, and Smad4, while improving the expression of Smad7 (Zhang et al., 2018). Chrysophanol, is an anthraquinone isolated from *Rheum officinale*, was administered to mice models of UUO and treatment with TGF-β1- at doses of 10, 20, and 40 mg/kg for 2 weeks stimulated renal tubular epithelial cells (Xie et al., 2019). This treatment regimen resulted in the upregulation of Smad7 expression, leading to amelioration of RF. This effect was achieved by inhibiting the expression of TGF-β1 and p-Smad3 expression, as well as disrupting the interaction between Smad3 and TβRI (Dou et al., 2020).

#### 4.2.5 Blood-activating TCM

The pharmacological effects of blood-activating TCM are manifested primarily in their regulatory effects on the blood system, such as dilating peripheral blood vessels to increase blood flow and reduce vascular resistance, inhibiting platelet aggregation, increasing plasmin activity to inhibit thrombosis, improving microblood flow to promote blood flow, relieving microvascular spasm and reducing capillary permeability (Fan et al., 2023; Yang et al., 2024). In RF, renal hemodynamics presents a high concentration, viscosity, coagulation, and aggregation state, which can easily lead to internal stasis stasis (Wolf, 1998). The addition of blood activating and blood stasis to TCM in the treatment process can improve hemodynamics and prevent thrombosis, which is consistent with the application of anti-coagulation drugs and anti-platelet aggregation in modern medicine. This demonstrates that medical research involving TCM follows the modern trend of drug research and will be an important approach for the treatment of diseases in the future.

*Salvia miltiorrhiza* Bunge, is called as “Dan Shen” in China, activates the blood to regulate menstruation, remove blood stasis and pain relief, cool blood to eliminate carbonic abscess, and eliminate restfulness and calm (Wang L. et al., 2023). Its main blood-activating component Tanshinone IIA is commonly used in the treatment of cardiovascular diseases. In recent years, they have shown promising results in the treatment of CKD (Wu S. et al., 2023; Cai et al., 2024). Administration of *Salvia miltiorrhiza* Bunge injection may alleviate streptozotocin (STZ) induced diabetic nephropathy, potentially by inhibiting oxidative stress, inflammatory reactions, and progression of fibrosis (Xu et al., 2016; Xu et al., 2020). *Curcuma longa* L. (Kotha and Luthria, 2019) suppressed the TGF-β/Smad signaling pathway and inhibited the expression of repulsive guidance molecule B and fibrogenic molecules in both rats and HK-2 cells (Chen F. et al., 2021).

### 4.3 Traditional Chinese Medicine compound targeting the TGF-β/Smad signaling pathway to prevent and treat renal fibrosis

Experimental studies on TCM and its effective extracts have provided a strong basis for the study of TCM compounds. The approach most frequently used in TCM for preventing and treating chronic renal failure and RF is the use of Chinese medicine compound preparations, which have shown positive efficacy (Table 2). TCM compounds can holistically regulate the renal microenvironment in clinical practice, and improve renal function with less side effects. Based on the patient’s specific symptoms and constitution, practitioners adjust the composition and dosage of the formula to achieve individualized treatment. From a mechanistic perspective, numerous TCM compounds are being investigated for their ability to target and regulate the TGF-β/Smad signaling pathway in the prevention and treatment of RF.

**TABLE 2:** Chinese herbal compound targeting the TGF-β/Smad signaling pathway for the prevention and treatment of renal fibrosis.

Name	Active ingredients	Dosage	Model	Mechanism	References
Liuwei Dihuang Pil	Herb couple *Rehmannia glutinosa* Libosch and *Cornus officinalis* Sieb	1 g/mL concentrated liquid prepared to meet the administration dose of 4 mL/kg	CKD model induced by adenine	Improving ECM components deposition, and diminish EMT, reducing the release of inflammatory cytokines and inhibits the TGF-β1/MAPK signaling pathway	Zhu et al. (2024)
Yi Shen Pai Du Formula	*Astragali radix*, *Rhei radix etrhizome*, *Hirudo*, *Bombyx batrytocatus*	2 g/kg/d	db/db mice, a model of type 2 diabetes that develops DKD	Inhibiting epithelial-to-mesenchymal transition (EMT) via suppression of the TGF-β/Smad pathway	Zhang Q. et al. (2021)
Shenkang VII Recipe	*Rhizoma Atractylod*, *Phellodendron bark*, *Smilax glabra*, *Rumex nepalensis Spreng*, *Bolbostemma paniculatum*, *Achyranthes* *Aspera*, *Astragalus membranaceus*, *Rhodiola rosea*	0.5, 1.0, or 2.0 g/kg/d	UUO in rats	Regulating ECM synthesis and degradation, reducing inflammation, and inhibiting fibroblast proliferation through the blockade of TGF-β1/Smad, NF-κB, and SHH signaling pathways	Zhou et al. (2020)
Kangxianling Decoction	*Rhizoma Atractylod*, *Phellodendron bark*, *Smilax glabra*, *Rumex nepalensis Spreng*, *Bolbostemma paniculatum*, *Achyranthes* *Aspera*, *Astragalus membranaceus*, *Rhodiola rosea*	0.5, 1.0, 2.0 g/kg/d	5/6 nephrectomy model	Downregulate the expression of TGF-β1, Smad2/3, CTGF, Collagen I, and Collagen III, and upregulate the expression level of Smad7	Ji and He, (2019)
Kangxianling Decoction	*Radix salvia miltiorrhizae*, *Rhubarb*, *Peach kernel*, *Radix angelicae ainensis*, *Badix achyranthis bidentatae*	21 g/kg/d	5/6 nephrectomy model	Inhibiting the production of ECM proteins and JNK, along with downregulation of TGF-β, Ang II	Ji et al. (2019)
Qingxuan Jiangya Decoction	*Gastrodia elata*, *Uncaria rhynchophylla*, *Chrysanthemum morifolium*, *Prunella vulgaris*, *Haliotidis Concha*, *Achyranthes bidentata*, *Eucommia ulmoides*, *Scutellaria baicalensis*, *Paeonia lactiflora*, *Glycyrrhiza uralensis*	60 mg/kg/d	spontaneously hypertensive rats	Inhibition of TGF-β/Smad signaling pathway	Liu et al. (2017)
Shenqi detoxification granule	*Astragalus membranaceus (Fisch.) Bge*, *Salvia miltiorrhiza Bge*, *Angelica sinensis* (Oliv.) Diels, *Rheum officinale Baill*, *Euryale ferox Salisb*, *Eclipta prostrata L*, *Eclipta prostrata L*, *Semiaquilegia adoxoides (DC.) Makino*, *Imperata cylindrica Beauv*. *var. major (Nees) C. E. Hubb*, *Aconitum carmichaelii Debx*, *Epimedium brevicornu Maxim*, *Rosa laevigata Michx*, *Hedyotis diffusa Willd*	2 mL	UUO in rats	Involving TGF-β1-mediated EMT and the TGF-β1-Smad-ILK signaling pathway	Cai et al. (2017)
Modified Huangqi Chifeng Decoction	*Astragalus membranaceus* (Fisch.) Bunge, *Paeonia veitchii Lynch*, *Saposhnikovia divaricata*	6.25, 12.5, 25.9 g/kg	An IgA nephropathy rat model	It reduced TGF-β1 expression in urinary exosomes and reduced TGF-β1 and p-Smad3 levels in renal tissues	Zhao M. et al. (2022)
Xiexin decoction	*Rheum palmatum*, *Coptis chinensis*, *Scutellaria baicalensis*	300, 600 mg/kg	db/db diabetic mice	Inhibiting the NF-κB and TGF-β/Smad pathways	Wu et al. (2015)
Jieduquyuzishen Prescription	*Artemisia annua* L., *Cimicifuga heracleifolia* Kom., *Hedyotis diffusa Willd*, *Paeonia suffruticosa Andr.*, *Trionyx sinensis Wiegmann*, *Centella asiatica* (L.) Urb., *Citrus medica L.* var. *sarcodactylis* *Swingle*, *Glycyrrhiza uralensis Fisch*, *Paeonia lactiflora Pall.*, *Rehmannia glutinosa Libosch*	18 mL/kg	MRL/lpr Mice	Inhibiting EMT and TGF- β 1/Smad2/3 Pathway	Wu et al. (2022)

The Yi Shen Pai Du Formula (YSPDF) is a TCM blend that has been utilized for over two decades for the clinical treatment of CKD. YSPDF mainly uses *Astragalus mongholicus* Bunge (Huang Qi), *Atractylodes macrocephala* Koidz. (Bai Zhu) and *Codonopsis pilosula* (Dang Shen) to supplement spleen and kidney, uses *Salvia miltiorrhiza* Bunge (Dan Shen) and *Leonurus japonicus* Houttuyn (Yi Mu Cao) to activate blood stasis, uses *Rheum officinale* (Da Huang) to remove blood stasis and diuresis to relieve turbidity (Zhang Y. et al., 2021). Overall, YSPDF can improve renal circulation, protect renal function, delay the process of renal failure effect. One study examined the effects of YSPDF on db/db mice, a model of type 2 diabetes and diabetic kidney disease (DKD). The study found that YSPDF notably enhanced various biochemical factors, reduced oxidative stress, and decreased levels of inflammatory cytokines by suppressing the TGF-β/Smad signaling pathway.

The Shenkang VII formula (SK-7) is a TCM blend that includes *Rhizoma Atractylodis* (Cang Zhu), *Phellodendron* (Huang Bo), *Smilax glabra* Roxb. (Tu Fu Ling), *Rheum officinale* (Da Huang), *Bolbostemma paniculatum* (Tu Bei Mu), *Achyranthes bidentata* Blume (Tu Niu Xi), *Astragalus mongholicus* Bunge (Huang Qi), and *Rhodiola rosea* L. (Hong Jing Tian). Some of their main components can provide kidney protection (Hao et al., 2022). This formula is typically consumed as a decoction and is commonly used in patients with CKD (Zhou et al., 2020). SK-7 controlled the production and degradation of ECM, decreased inflammation by inhibiting the TGF-β/Smad pathway and demonstrated inhibitory effects on RF in UUO model rats.

Professor He Liqun of Shuguang Hospital Affiliated to the Shanghai University of Traditional Chinese medicine conducted a multi-center clinical controlled sample observational study of patients with RF, and found that blood stasis runs throughout the CKD process. Therefore, the treatment of blood circulation and blood stasis should be considered during the pathogenesis of RF. We developed a Kangxianling (KXL) decoction consisting of *Salvia miltiorrhiza* Bunge (Dan Shen), *Rheum officinale* (Da Huang), peach kernel (Tao Ren), *Angelica sinensis* (Dang Gui), and *Achyranthes bidentata* Blume (Niu Xi) (Zhang et al., 2007; He et al., 2009; Ji et al., 2019). KXL has the potential to decrease RF by suppressing the synthesis of ECM proteins and c-Jun N-terminal kinase (JNK) expression, as well as by reducing the expression of TGF-β and Ang II in a rat model of 5/6 Nephrectomy (5/6 N) *in vivo* and in Ang II-treated rat glomerular mesangial cells *in vitro* (Ji et al., 2019). The administration of KXL may reduce renal histopathological damage and modulate the expression of various proteins involved in fibrosis, such as TGF-β1, Smad2/3, connective tissue growth factor (CTGF), collagen I, and collagen III, while concurrently increasing the expression of Smad7 (Ji and He, 2019). Overall, KXL can alleviate RF by enhancing blood flow and eliminating stagnant blood, restoring the functions of the spleen and kidney, boosting the vital energy of the body, clearing the intestines, purifying turbidity, promoting toxin excretion, improving kidney edema, improving immunity, improving hemodynamic disorders, exerting anti-inflammatory effects, reducing ureteral obstruction, and regulating blood lipid metabolism.

Qingxuan Jiangya Decoction (QXJYD) was developed by the academician Chen Keji, with more than 60 years of clinical experience in prescription. The prescription of Jun medicine can calm the liver and extinguish the wind of *Gastrodia elata* Blume (Tian Ma), Hook vine, Kuding tea, *Eucommia ulmoides* Oliv (Du Zhong). The medicine can nourish the liver and kidney, with the effect of calming the nerves. Night sex vine can clear the liver heat. *Chrysanthemum indicum* L., Mulberry leaves, and *Scutellaria baicalensis* Georgi (Huang Qin) are also a part of the formula, as well as *Rehmannia glutinosa* (Di Huang) that nourishes yin and clears heat. The combination of various medications can achieve the effect of clearing liver heat, flattening liver yang, and benefiting the liver and kidney (He et al., 2020). Clinical studies have shown that QXJYD has superior antihypertensive effects in patients with hypertension (Huang et al., 2019; Zhang et al., 2020). Some studies had found that in addition to hypertension, QXJYT could significantly reduce the appearance of renal interstitial fibrosis in the spontaneous hypertension rat (SHR) model, and the antihypertensive and nephroprotective effects of QXJYT were found to be significantly associated with the suppression of the TGF-β/Smad signaling pathway (Liu et al., 2017).

Shenqi detoxification granule (SDG), an ancient Chinese herbal remedy, have been used for many years to treat CKD in clinical practice with the function of tonifying the kidney, invigorating blood circulation and detoxifcation (Peng et al., 2014; Cai et al., 2017). SDG contains 12 herbs: *Astragalus mongholicus* Bunge (Huang Qi), *Salvia miltiorrhiza* Bunge (Dan Shen), *Angelica sinensis* (Dang Gui), *Rheum officinale* (Da Huang), and *Euryale ferox* Salisb (Qian Shi). Treatment with SDG and P311 improves renal function in rats with UUO, and this effect might be associated with the process of EMT mediated by TGF-β1 (Cai et al., 2017).

The modified Huangqi Chifeng Decoction (MHCD) contains *Astragalus mongholicus* Bunge (Huang Qi), *Paeonia lactiflora* Pall (Sheng Chishao) and *Saposhnikoviae* Radix (Fang Feng), and has the effect of invigorating qi and activating blood, clearing collaterals, and opening orifices. MHCD could restrain inflammatory factors that stimulate the secretion of ECM in glomerular mesangial cells, as well as suppress excessive activation of the TGF-β/Smad signaling pathway (Gao et al., 2016). Xiexin decoction (XXD) is a TCM formula that has been used for more than 1,300 years for the treatment of diabetes. This prescription comprises Radix et *Rheum officinale* (Da Huang), *Coptis chinensis* (Huang Lian), and *Scutellaria baicalensis* Georgi (Huang Qin) as the key components (Wu et al., 2015; Wang H. et al., 2023). It has the ability to regulate the digestive, absorption, and metabolic processes of the gastrointestinal system (Liao et al., 2023). The active components of XXD in combination were found to reduced RF in diabetic nephropathy by targeting the NF-κB and TGF-β/Smad signaling pathways (Wu et al., 2015). The Jieduquyuzishen prescription (JP) has been used extensively in China for the treatment of lupus nephritis, demonstrating favorable clinical outcomes (Ye et al., 1994). JP consists of a combination of ten different herbs, including *Artemisia annua* L. among others (Wu S. et al., 2022). JP can reduce RF in MRL/lpr mice by suppressing EMT and TGF-β/Smad2/3 signaling pathway (Wu S. et al., 2022). Multi-component herbal medicine may be an effective method to treat diabetic nephropathy.

## 5 Discussion and future perspectives

**FIGURE 3:**
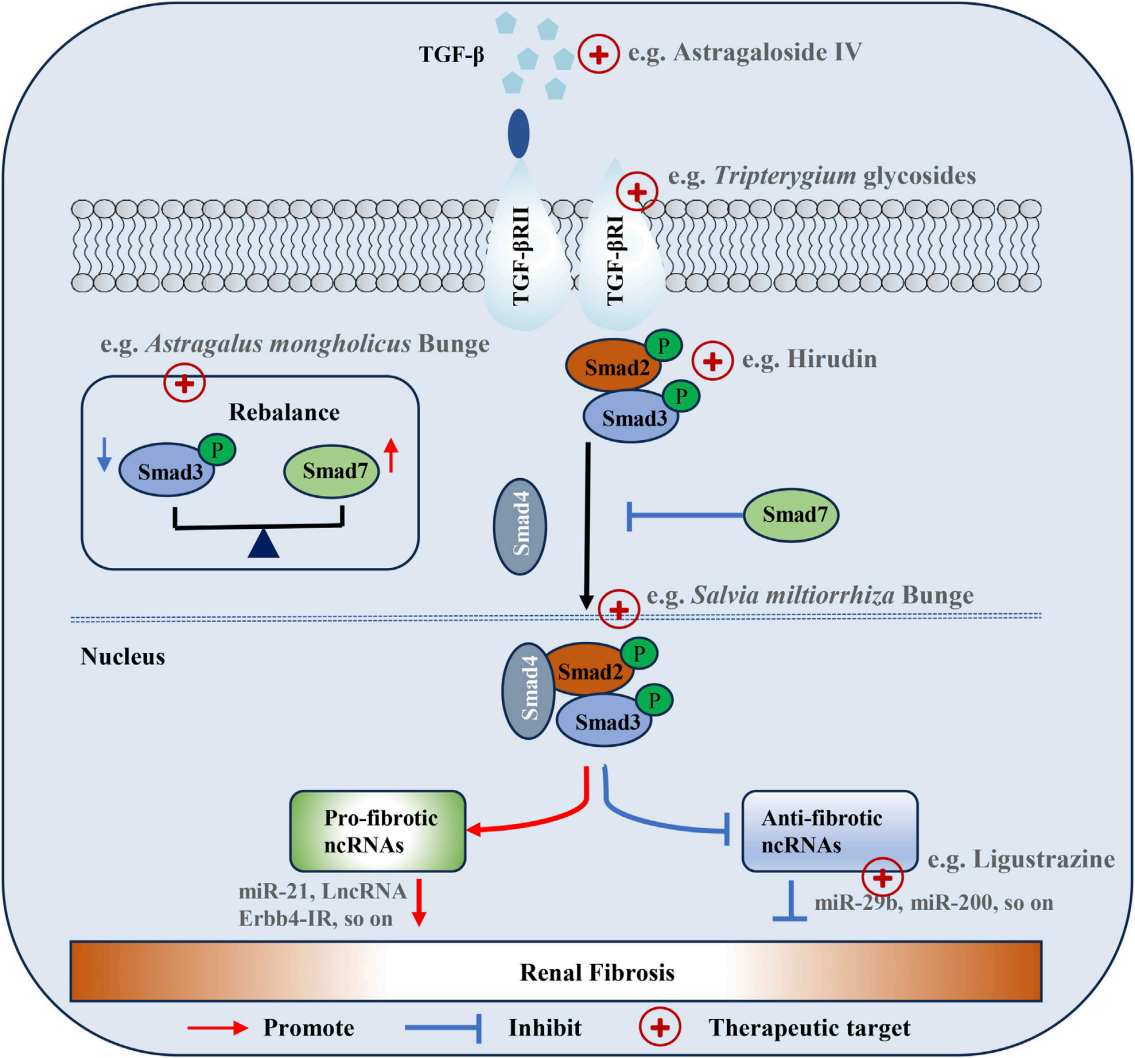
Therapeutic targets in TGF-β/Smad signaling in renal fibrosis.

TCM has unique advantages in the prevention, treatment, and rehabilitation of diseases. Numerous research studies have shown that the natural components of TCM exhibit antioxidant and anti-inflammatory properties, which contributes to its efficacy in mitigating RF (Wei et al., 2002; Zhou et al., 2022; Song Z. et al., 2024; Xiang et al., 2024). The objective of our review was to examine the mechanism and function of TCM in relation to the TGF-β/Smad signaling pathway in RF (Liu X. J. et al., 2022). This review provides a detailed analysis and synthesis of available information to present new perspectives and potential therapeutic targets in RF. Current research suggests that Chinese herbal medicine, particularly compound preparations of TCM, offers the benefit of a multi-target intervention to improve RF and presents extensive avenues for future investigation. On one hand, TCM can inhibit the expression and activation of TGF-β, including reducing its secretion and blocking the activation process, thus controlling its ability to activate the signaling pathway from the source. On the other hand, it can regulate the activity and expression of Smad proteins. By inhibiting the phosphorylation of Smad2 and Smad3, as well as regulating the expression levels of Smad3 and Smad7, etc., TCM can block signal transduction and reduce the synthesis of ECM (Figure 3). TCM can also affect the upstream and downstream related signaling molecules and pathways. For example, it can regulate miRNAs to indirectly act on this signaling pathway, or interfere with other cross-linked signaling pathways to regulate it indirectly. At the same time, the anti-inflammatory and anti-oxidant effects of TCM should not be underestimated. It can reduce the release of inflammatory mediators and inhibit the activation of the TGF-β/Smad signaling pathway induced by inflammation. Additionally, it can scavenge ROS and alleviate oxidative stress damage to inhibit this signaling pathway, thereby protecting renal cells and delaying the progression of RF. However, most Chinese herbal medicine interventions for RF have focused on their overall effectiveness, whereas it is imperative to conduct in-depth investigations into the pharmacological constituents and mechanisms of action of individual ingredients.

Research shows that the dosage selection and treatment duration of active components in TCM (such as Astragaloside IV, emodin, and tripterygium glycosides) significantly influence their anti-fibrotic efficacy. Based on animal models and pharmacokinetic studies, most components exhibit a “bell-shaped” dose-response curve. For example, Astragaloside IV can optimally inhibit the phosphorylation of Smad2/3 within the dosage range of 40–60 mg/kg, while the risk of hepatotoxicity increases when the dosage of tripterygium glycosides exceeds 100 mg/kg (Wan et al., 2014; Mao et al., 2019). Clinical observations have also documented cases of reproductive system impairment in patients following prolonged triptolide use (Song Z. et al., 2024). In terms of treatment duration, short-term intervention (1–2 weeks) mainly regulates the secretion of TGF-β1, while continuous administration for 4–8 weeks is required to improve structural fibrosis (Dou et al., 2020). It should be noted that certain TCM carry potential side effects and toxicological risks, which become particularly pronounced during long-term administration or at high dosages. The complex composition of compound TCM formulations may lead to unpredictable toxicological interactions between various active constituents. Current clinical evidence supports the anti-renal fibrotic potential of TCM, though their therapeutic efficacy appears more moderate than preclinical observations suggest, and their safety profiles warrant further rigorous evaluation. There is a relative paucity of large-scale, long-term clinical safety studies on these herbal components, particularly regarding the chronic safety profile of multi-herb formulations where the combined effects of multiple active compounds remain to be fully elucidated.

TCM and modern pharmacological therapies offer distinct yet complementary approaches to treating RF. Modern pharmacological therapies, such as ACEI/ARBs, SGLT2 inhibitors, and antifibrotic drugs, provide precise, fast-acting interventions supported by robust clinical trials (Huang et al., 2024), suitable for middle and late-stage patients, but potential side effects and economic burdens may exist. In contrast, TCM, characterized by its multi-component and multi-target mechanisms, emphasizes holistic regulation and long-term management with fewer side effects, making it suitable for early-stage CKD and patients with systemic symptoms but takes longer to show effects (Liu X. Y. et al., 2022). However, its complex composition and lack of standardized clinical evidence pose challenges. Given their respective advantages and disadvantages, combined treatment of TCM and modern pharmacology therapies can exert a synergistic effect, leveraging the strengths of both to improve outcomes in RF treatment.

While TCM shows potential in treating RF through its multi-target and holistic approach, it faces significant limitations, including unclear mechanisms of action, challenges in quality control and standardization, insufficient high-quality clinical evidence, regulatory hurdles, and cultural barriers to international acceptance (Song L. et al., 2024; Li and Xiao, 2025). Regulatory frameworks for TCM are often underdeveloped, with complex approval processes that struggle to accommodate the multi-component nature of herbal formulations. Standardization of TCM products faces significant challenges due to variations in raw materials and processing methods, resulting in inconsistent batch-to-batch concentrations of active ingredients. For example, the content of Astragaloside IV in Astragalus membranaceus from different production areas and harvest seasons can vary by up to 30%. This requires the implementation of standardization through HPLC fingerprinting combined with bioactivity testing. Additionally, the lack of high-quality clinical evidence and unclear mechanisms of action hinder international acceptance and integration into modern medical practices. At the same time, potential side effects, drug interactions, and the complexity of individualized treatment further limit its widespread application. Addressing these limitations requires advancing research, improving standardization, conducting rigorous clinical trials, and fostering international collaboration to integrate TCM into modern medical practices for the treatment of RF.

Future research on TCM for RF should focus on elucidating mechanisms of action through advanced technologies like network pharmacology and metabolomics, improving quality control and standardization of herbal products, conducting high-quality clinical trials with modern biomarkers, and enhancing regulatory frameworks to support international acceptance. Additionally, exploring TCM-Western drug combination therapies, fostering global collaboration, and developing modern TCM formulations (e.g., nanotechnology-based delivery systems) are essential to advance the scientific and clinical integration of TCM in RF treatment. These efforts will help overcome current limitations and promote the global application of TCM in modern medicine.
