# Supplementary material for: Traditional Chinese Medicine targeting the TGF-β/Smad signaling pathway as a potential therapeutic strategy for renal fibrosis
Source: Front Pharmacol. 2025 May 20;16:1513329. doi: 10.3389/fphar.2025.1513329 (PMC12130906; doi:10.3389/fphar.2025.1513329)
Supplement: Supplementary file 1 [file DataSheet1.pdf]

An assessment of the literature

1. Mudan granules

Assessment Item	Specific Content
Description of Plant Material	Composed of Huangqi (*Astragalus membranaceus*), Yanhusuo (*Corydalis yanhusuo*), Sanqi (*Panax notoginseng*), Chishao (*Paeoniae Radix Rubra*), Danshen (*Salvia miltiorrhiza* Bunge), Chuanxiong (*Ligusticum chuanxiong* Hort.), Honghua (*Carthamus tinctorius*), Sumu (*Haematoxylum campechianum*), Jixueteng (*Spatholobus suberectus Dunn*)
Extraction Method	Mudan granules were provided by Liaoning Oda Pharmaceutical Co., Ltd (batch number: 10211019, Liaoning, China)
Chemical Analysis	Mudan granules have been widely used in clinical practice, therefore, no chemical analysis was carried out in this paper.
Experimental Design	Type I Diabetes mellitus (T1DM) model was used, with a dosage of 1.08,2.16 and 4.32 g/kg, administered daily by gavage for 12 consecutive weeks.
Data Analysis	Statistical analysis was performed using one-way analysis of variances, with results expressed as mean ± standard deviation.
Ethical Compliance	The experiment was approved by the Institutional Animal Ethics Committee (Approval No.: 2022030502).

2. Astragaloside IV (ASI)

Assessment Item	Specific Content
Description of Plant Material	ASI is the major active constituent of *Astragalus membranaceus*.
Extraction Method	ASI was purchased from Shanghai Jizhi Biochemical Technology Co., Ltd. (cat. no. A50670; purity ≥99%).
Chemical Analysis	HPLC was used to determine the content of ASI, and a chemical fingerprint was established.
Experimental Design	DN rat model was used, with a dosage of 40 mg/kg, administered daily by gavage for 8 weeks.
Data Analysis	Statistically significant differences between groups were determined using a Student's t-test. Multiple comparisons were made among three or more groups using one-way ANOVA followed by the Bonferroni post-hoc test. The nonparametric Mann-Whitney U test was used if data were not normally distributed. All results are presented as the mean ± standard deviation.
Ethical Compliance	The experiment was approved by the Institutional Animal Ethics Committee (Approval No.: Y20170623).

3. *Coreopsis tinctoria* Nutt

Assessment Item	Specific Content
Description of Plant Material	*Coreopsis tinctoria* Nutt is a plant of the genus Asteraceae in the family of Compositae.
Extraction Method	C. tinctoria Nutt was harvested from Minfeng county, Hetian city in Xinjiang province, China. The dried flowers of C. tinctoria Nutt (160 g) were ground into powder and placed in 4 L of 55 % ethanol for reflux extraction twice at 80 °C for 2 h. The extracting liquid was filtered, merged, and then concentrated by rotary evaporator (R-210; Buchi, Essen, Germany) into 1 L liquid extract. An equal volume of AcOEt was added into the 1 L of liquid extract and then was concentrated and spray dried to obtain the powder of AC (4.4 %, w/w:dry flower). The powder of AC and metformin were weighed and dissolved in distilled water containing 1 % (g/100 mL) sodium carboxymethyl cellulose and ultrasonicated for 4 h until the powder was completely dissolved.
Chemical Analysis	HPLC was used to determine the content of AC, and a chemical fingerprint was established.
Experimental Design	A diabetic rat model was induced by high-glucose–fat diet and intraperitoneal injection of 35 mg/kg STZ. Diabetic rats with 150, 300 and 600 mg/kg AC per day, respectively, by gavage administration for 4 weeks.
Data Analysis	Differences between multiple groups were analysed by one-way analysis of variance followed by Duncan’s multiple range test. Differences between two groups were measured by Student’s t test. All data are presented as the mean ± SEM.
Ethical Compliance	The experiment was approved by the Institutional Animal Ethics Committee (Approval No.: IACUC-20140304011).

4. *Astragalus* polysaccharides

Assessment Item	Specific Content
Description of Plant Material	It is derived from a leguminous plant, *Astragalus propinquus*.
Extraction Method	*Astragalus* polysaccharides (purity >98%) were from Lanzhou Wotelaisi Biological Co. Ltd. (Lanzhou, China).
Chemical Analysis	No chemical analysis was carried out in this paper.
Experimental Design	A type 2 diabetic rat model was established with a high-fat diet in combination with low-dose streptozotocin (35 mg/kg), given 25, 50, and 100 mg/kg of *Astragalus* polysaccharides, respectively, by intragastric administration once daily.
Data Analysis	Measurement data were expressed as mean ± standard deviation and compared with one-way analysis of variance (ANOVA) and q test.
Ethical Compliance	The experiment was approved by the Institutional Animal Ethics Committee (Approval No.: IACUC: 1804021).

5. Alpha-mangostin

Assessment Item	Specific Content
Description of Plant Material	It is derived from a leguminous plant, *Astragalus propinquus*.
Extraction Method	The α-mangostin (α-Man) was purchased from Chengdu Biopurify Phytochemical Ltd (Sichuan, China).
Chemical Analysis	No chemical analysis was carried out in this paper.
Experimental Design	UUO model was used, with a dosage of 10 and 20 mg/kg, oral gavage for 7 days.
Data Analysis	All data are expressed as mean ± SD. Statistical significance was analyzed using one-way analysis of variance (ANOVA).
Ethical Compliance	The experiment was approved by the Institutional Animal Ethics Committee (IACUC approval code: 2585).

6. Baicalin and baicalein

Assessment Item	Specific Content
Description of Plant Material	Baicalin and baicalein are flavonoid compounds derived from *Scutellaria baicalensis Georgi*. T.
Extraction Method	They were purchased from National Institute for the Control of Pharmaceutical and Biological Products, Beijing, China)
Chemical Analysis	No chemical analysis was carried out in this paper.
Experimental Design	TGF-β1-induced in vitro model of fibrosis, incubated with or without baicalin/baicalein (20, 40, 80 μM) for 48 h.
Data Analysis	All data are expressed as mean ± SEM. The statistical significances of differences between groups were analyzed via one-way analysis of the variance followed by a post hoc Newman-Keuls test.
Ethical Compliance	This article does not involve animal experiments.

7. Baicalin

Assessment Item	Specific Content
Description of Plant Material	Baicalin, a flavonoid extracted from radix scutellariae.
Extraction Method	Baicalin (HPLC ± 98%, batch number: SB8020, Solarbio Life Sciences, Beijing, China).
Chemical Analysis	No chemical analysis was carried out in this paper.
Experimental Design	UUO RIF model was used, with a dosage of 10, 20 and 40 mg/kg, gavage once a day for a total of 14 days.
Data Analysis	All data are expressed as mean ± SD. Differences between groups were compared by one-way ANOVA and Tukey’s post hoc test.
Ethical Compliance	The experiment was approved by the Institutional Animal Ethics Committee (Approval: NXLL-007-A).

8. Wogonin

Assessment Item	Specific Content
Description of Plant Material	It is major components of *Scutellaria baicalensis Georgi*.
Extraction Method	wogonin （Meilun Biology Technology, Da lian, China）
Chemical Analysis	No chemical analysis was carried out in this paper.
Experimental Design	TGF-β1 treated HK-2 cells model was used, with a treatment of 1, 2 and 4 μg/μl.
Data Analysis	Data are expressed as the mean ± SD and analyzed using Kruskal-Wallis one-way analysis of variance (ANOVA).
Ethical Compliance	This article does not involve animal experiments.

9. *Glycyrrhiza uralensis* root extract

Assessment Item	Specific Content
Description of Plant Material	Roots of *Glycyrrhiza uralensis* (*Radix glycyrrhizae*) used as a sweetener and traditional Chinese medicine possess high potential for renal protection.
Extraction Method	The aqueous extract was prepared by heating 200 grams of *R. glycyrrhizae* root with 1000 ml of distilled water and evaporated to 100 ml.
Chemical Analysis	HPLC-MS/MS was used to determine the content, and a chemical fingerprint was established.
Experimental Design	Apoe$^{em1/Narl}$/Narl male mice model was used, with a dosage of 100, 150 and 200 μg/kg, given orally for 8 weeks.
Data Analysis	All data are expressed as mean ± SE. Statistical analysis was performed by multiple comparison tests with one--way analysis of variance (ANOVA) followed by Tukey test.
Ethical Compliance	The experiment was approved by the Institutional Animal Ethics Committee (Approval: CMUBH R104--005).

9. Amygdalin

Assessment Item	Specific Content
Description of Plant Material	Amygdalin is a hydroglycoside widely found in the seeds of Rosaceae plants, such as peach, apricot, and plum.
Extraction Method	Amygdalin (A6005) was brought from Sigma company (St Louis, America)
Chemical Analysis	No chemical analysis was carried out in this paper.
Experimental Design	Streptozotocin (STZ)-induced diabetic nephropathy (DN) rat model was used, with a dosage of 1, 3 and 10 mg/kg/day, given orally for 8 weeks.
Data Analysis	All data are expressed as mean ± SD. Two-factor analysis of variance was used for blood glucose data. One-way analysis of variance was applied for pairwise comparison at each time.
Ethical Compliance	The experiment was approved by the Institutional Animal Ethics Committee (Approval: ACU2019031212CU).

10. *Cordyceps cicadae* polysaccharides (CCP)

Assessment Item	Specific Content
Description of Plant Material	A class of polysaccharide substances extracted from *Cordyceps cicadae*.
Extraction Method	Dried powder of Cordyceps cicadae (100 g) was dipped into distilled water (1:10, w/v) three times at 90°C for six hours. The supernatant was concentrated to 80 mL with a rotary evaporator at 65°C under a vacuum. Then, the concentrate was added to 95% (v/v) ethanol in a ratio of 1:4 and kept overnight at 4°C. The precipitates were obtained by centrifugation (5000 rpm for 10 minutes) and dissolved in distilled water to remove proteins using the Sevag method (chloroform: butanol in a ratio of 4:1).
Chemical Analysis	The monosaccharide compositions of CCP were determined by high performance liquid chromatography equipped with a DAD detector at 245 nm and Zorbox Eclipse XDB-C18, and a chemical fingerprint was established.
Experimental Design	SD rats fed with a high-fat diet model was used, with a dosage of 75, 150 and 300 mg/kg, gavaged for 4 consecutive weeks.
Data Analysis	All data are expressed as mean ± SD. SPSS version 17.0 service solution software (SPSS Inc., USA) was used for statistical analysis of the statistical significance of the differences between different groups.
Ethical Compliance	The experiment was approved by the Institutional Animal Ethics Committee (Approval: 2019-002).

11. Acetylshikonin

Assessment Item	Specific Content
Description of Plant Material	The main ingredient of Zicao, a well-known Chinese traditional medicine.
Extraction Method	Acetylshikonin (≥ 98.0% purity assayed by HPLC) was purchased from Shifeng Biological Technology (Shanghai, China).
Chemical Analysis	No chemical analysis was carried out in this paper.
Experimental Design	Diabetes with STZ model was used, were intragastric administration with 100 mg/kg/day acetylshikonin for 8 weeks.
Data Analysis	All data are expressed as mean ± SEM. Statistical analysis of data from two groups was performed using two-tailed Student t test. Data from more than two groups were compared by one-way ANOVA, followed by the Bonferroni multiple comparison test.
Ethical Compliance	All experimental protocols for animals were carried out in strict accordance with the Institutional Animal Care and Use Committee of Cangzhou Central Hospital.

12. *Cordyceps sinensis*

Assessment Item	Specific Content
Description of Plant Material	The traditional medicine known as Chinese caterpillar fungus (Dongchong Xiacao) is composed of the parasitic fungus Cordyceps sinensis, which grows on the larva of the ghost moth Hepialus armoricanus Oberthur (Hepiallidae).
Extraction Method	Artificially cultured *C. sinensis* was kindly provided by Hangzhou Huadong Pharmaceutical Company (Hangzhou, China).
Chemical Analysis	No chemical analysis was carried out in this paper.
Experimental Design	5/6 nephrectomy model was used, with 2.0 g/kg d by intragastric administration for 12 weeks.
Data Analysis	All data are expressed as mean ± SD. One-way analysis of variance (ANOVA) and Mann-Whitney U analysis were used to perform comparisons among the different groups.
Ethical Compliance	All animal experiments were performed with the approval of the Ethical Committee of Southeast University.

13. Isoliquiritigenin

Assessment Item	Specific Content
Description of Plant Material	A chemical component extracted from *Glycyrrhiza uralensis Fish*.
Extraction Method	Isoliquiritigenin was provided by Sigma Chemical (St. Louis, MO).
Chemical Analysis	No chemical analysis was carried out in this paper.
Experimental Design	Human mesangial cells model was used, with 1, 10 and 20 μM treated.
Data Analysis	All data are expressed as mean ± SEM. Statistical analyses were conducted using Statistical Analysis Systems statistical software package (SAS Institute, Cary, NC). Significance was determined by one-way ANOVA.
Ethical Compliance	This article does not involve animal experiments.

14. Oxymatrine (OM)

Assessment Item	Specific Content
Description of Plant Material	Oxymatrine is an herbal product derived from the root of Sophora flavescens Ait
Extraction Method	OM was purchased from the China National Institute of Control of Pharmaceutical and Biological Products (Batch Number: 110780–201007).
Chemical Analysis	No chemical analysis was carried out in this paper.
Experimental Design	Rat renal tubular epithelial cells (NRK52Es) model was used, with 0.5 mg/ml treated.
Data Analysis	All data are expressed as mean ± SD. One-way ANOVA and Student’s Newman-Keuls test for comparisons were used to determine differences between control and experimental groups. Student's t-test was performed for paired samples.
Ethical Compliance	This article does not involve animal experiments.

15. Salidroside

Assessment Item	Specific Content
Description of Plant Material	Oxymatrine is an herbal product derived from the root of Sophora flavescens Ait
Extraction Method	Salidroside (≥ 98.0% purity assayed by HPLC) was purchased from Sigma Chemical (St. Louis, MO).
Chemical Analysis	No chemical analysis was carried out in this paper.
Experimental Design	STZ combined with right nephrectomy model was used, with 50, 100 and 200 mg/kg/day, given orally for 12 weeks.
Data Analysis	All data are expressed as mean ± SD. One-way ANOVA and Student's Newman-Keuls test for comparisons were used to determine differences between control and experimental groups. Student's t-test was performed for paired samples.
Ethical Compliance	The experiment was approved by the Institutional Animal Ethics Committee (Approval: 20180769).

16. Levistolide A

Assessment Item	Specific Content
Description of Plant Material	A natural compound extracted from *Angelica sinensis* (Oliv.) Diels.
Extraction Method	The dried roots of A. sinensis (10 kg) were exhaustively extracted with 95% EtOH (45 L each time) under reflux three times. The combined solvents were removed under vacuum to afford a crude extract. The EtOH extract was suspended in water and partitioned successively with petroleum ether (PE) and ethyl acetate (EtOAc) to yield a PE extract (201 g) and an EtOAc extract (69.8 g). The PE extract was subjected to silica gel (300-400 mesh) column chromatography (P. ether-EtOAc 100:1, 50:1, 20:1, 10:1, 4:1, 1:1) to yield thirteen fractions A-M. Fraction D was subjected to silica gel column chromatography (P. ether-EtOAc 40:1, 20:1, 10:1, 5:1, 1:1) and yielded eight subfractions Fr.D.1~Fr.D.8. Subsequently, Fr.D.5 was separated by chromatography on Sephadex LH-20 gel (eluted with MeOH) to afford five subfractions Fr.D.5.1~Fr.D.5.5, and Fr.D.5.3 was subjected to silica gel column chromatography (P. ether-CH2Cl2 2:1) to give compound 10 (53.0 mg).
Chemical Analysis	HPLC-DAD was used to determine the content, and a chemical fingerprint was established.
Experimental Design	5/6 nephrectomy (Nx) mice model was used, with 0.5, 1.0 and 2.0 mg/kg/day, intraperitoneal injection every day for 4 weeks.
Data Analysis	All data are expressed as mean ± SEM. The statistical significance between two groups or more than three groups were evaluated using Student's t-test or one-way ANOVA with Tukey's test, respectively.
Ethical Compliance	The experiment was approved by the Institutional Animal Ethics Committee (Approval: NEU-EC-2021A002S).

17. Poricoic acid ZA (PZA)

Assessment Item	Specific Content
Description of Plant Material	PZA is a small compound isolated from layer of *P. cocos* (LPC).
Extraction Method	Pulverized LPC (20 kg) collected in Yunnan Province (China) was extracted three times in 95% EtOH (100 l). The solvent was removed under reduced pressure. The resulting residue (828 g) was subsequently dissolved in water and extracted with petroleum ether, EtOAC, and nBuOH for three times, respectively.
Chemical Analysis	No chemical fingerprint was established.
Experimental Design	HK-2 cells and podocytes induced by TGF-β1 and angiotensin II (ANGII) cells model was used, treated with different concentrations of PZA (0, 1, 10, 50, 100 μM) for 24-h.
Data Analysis	All data are expressed as mean ± SD. Comparison between groups were assessed by one-way ANOVA.
Ethical Compliance	This article does not involve animal experiments.

18. Sang-Bai-Pi extract

Assessment Item	Specific Content
Description of Plant Material	The root bark of *Morus alba L.* (*M. alba*), known as Sang-Bai-Pi in traditional Chinese medicine (TCM), is well-known for its medicinal use to relieve asthma by clearing lung heat (related to lung function) and to reduce edema by inducing diuresis (related to kidney function).
Extraction Method	The root barks of M. alba were peeled from the roots collected from the Mount Kunyu area in Shandong province, China. The EtOH extract, solvent partitions, and various fractions were prepared as described in a previous study. EA-1, EA-2, EA-3, and EA-4 represent the 30 %, 50 %, 80 %, and 95 % EtOH eluates, respectively, from the ethyl acetate partition fractionated by macroporous resin column chromatography, and n-Bu represents the n-BuOH partition.
Chemical Analysis	No chemical fingerprint was established.
Experimental Design	UUO mouse model was used, treated with different concentrations of 200 ang 300 mg/kg for 14 days.
Data Analysis	Statistical analysis was performed using GraphPad Prism software version 8.0.2 (GraphPad Software, San Diego, CA, USA) and one-way analysis of variance (ANOVA).
Ethical Compliance	The experiment was approved by the Institutional Animal Ethics Committee (Approval: 2023-030).

19. multi-glycoside of *Tripterygium wilfordii Hook. f.*

Assessment Item	Specific Content
Description of Plant Material	Multi-glycoside of Tripterygium wilfordii Hook. f. is a stable glycoside extracted from Tripterygium wilfordii Hook. f. (TWHF), also known as "Lei Gong Teng (the local name in China)".
Extraction Method	It was purchased by Jiangsu Meitong Pharmaceutical Co., Ltd. of Jiangsu Province (Taizhou, China).
Chemical Analysis	No chemical fingerprint was established.
Experimental Design	Adriamycin (ADR)-induced nephropathy (ADRN) model was used. Daily oral administration with the low dose at 50 mg/kg BW or the high dose at 100 mg/kg BW for 6 weeks.
Data Analysis	All data are expressed as mean ± SEM. We used the one-way analysis of variance (ANOVA) followed by Tukey test for multiple comparisons.
Ethical Compliance	The experimental protocol was approved by the Animal Ethics Committee of Nanjing University Medical School.

20. Celastrol

Assessment Item	Specific Content
Description of Plant Material	Celastrol ($C_{29}H_{38}O_4$) is a bioactive compound extracted from the traditional Chinese medicinal plant *Tripterygium wilfordii Hook F* (TwHF, thunder God Vine).
Extraction Method	It was purchased by MCE (Medchemexpress, NJ, USA).
Chemical Analysis	No chemical fingerprint was established.
Experimental Design	UUO model was used. The mice were treated with celastrol (1 mg/kg) by intraperitoneal injection for 7 days from the day of surgery.
Data Analysis	All data are expressed as mean ± SEM. Results from different groups were analyzed by Student's t-test or one-way analysis of variance (ANOVA) followed by Tukey's post hoc test.
Ethical Compliance	All animal studies were approved by the Institutional Animal Care and Use Committee of the Third Military Medical University.

20. Emodin

Assessment Item	Specific Content
Description of Plant Material	Emodin is a natural component extracted from Chinese herbs, such as *Cassia obtusifolia* and *Rheum palmatum*, and it exhibits a variety of biological activities including neuroprotective, anticancer, anti-infammatory, anti-allergic and hepatoprotective activities.
Extraction Method	Emodin was obtained from Shanghai Yuanye Bio-Technology (Shanghai, China).
Chemical Analysis	No chemical fingerprint was established.
Experimental Design	5/6 renal mass reduction model was used. The mice were treated with 0.3 and 1.0 mg/kg by intraperitoneal injection 10% chloral Hydrate for 7 days.
Data Analysis	All data are expressed as mean ± SD. Statistical analyses of data were performed by one-way analysis of variance (ANOVA) and Student’s t test using SPSS 20.0 software (SPSS Inc., USA).
Ethical Compliance	All animal studies were approved by the Institutional Animal Care and Use Committee of the Third Military Medical University.

21. Rhubarb extracts

Assessment Item	Specific Content
Description of Plant Material	Rhubarb, a well-known traditional Chinese medicine, has been widely used in China and Japan to treat CKD and reveals promising clinical prospects.
Extraction Method	Rhubarb was ground to powder and the powder was sieved by 20 meshes. Then rhubarb powder (2 kg) was extracted with 15 L 95% ethanol for 30 min by ultrasonic method for three times. The resulting extract was concentrated under pressure to yield a brown ethanol extract. The ethanol extract obtained was partitioned between water and three organic solvents with different polarities (BU> EA> PE), to yield three new fractions including PE, EA, and BU extracts.
Chemical Analysis	UPLC-MS was used to determine the content, but no chemical fingerprint was established.
Experimental Design	CKD rat model was used. They were administered PE extract (800 mg/kg), EA extract (200 mg/kg), and BU extract (600 mg/kg) by gastric irrigation, respectively, during 6 weeks study periods.
Data Analysis	The resulting p-values from Student’s t-test were further adjusted by a false discovery rate (FDR) based on the Benjamini–Hochberg method.
Ethical Compliance	This study was approved by the Ethical Committee of Northwest University and studies were performed in accordance with the Guide for the Care and Use of Laboratory Animals defined by Ethical Committee of Northwest University.

22. Chrysophanol

Assessment Item	Specific Content
Description of Plant Material	Chrysophanol (CP), 1,8-dihydroxy-3-methyl-9,10-anthraquinone, is an anthraquinone isolated from *Rheum palmatum L.* with a variety of pharmacological activities including the suppression of RIF.
Extraction Method	CP (≥ 98.0% purity, Cas: 481-74-3, Melonepharma, Dalian, China)
Chemical Analysis	No chemical fingerprint was established.
Experimental Design	UUO model was used. The mice were treated with 10 and 20 mg/kg and administered the suspension for 14 days by gavage, after completion of modeling.
Data Analysis	One-way ANOVA was used for analysing the differences between various experimental variables when there were more than two groups. Comparisons between two groups were made using a two-tailed unpaired Student's t-test
Ethical Compliance	The experiment was approved by the Institutional Animal Ethics Committee (Approval: XJ20170207).

23. Hirudin

Assessment Item	Specific Content
Description of Plant Material	Hirudin, an extract from salivary glands of medicinal leeches (*Hirudo medicinalis*), is one of the most potent natural inhibitors of thrombin.
Extraction Method	Hirudin (patent no. ZL03113566.8, 100 IU/vial, Canton Xike Kang Biotechnology Co., Ltd., Guangxi, China)
Chemical Analysis	No chemical fingerprint was established.
Experimental Design	UUO rat model was used. Three UUO + hirudin-treatment groups (10, 20, or 40 IU/kg/d, for 14 continuous days).
Data Analysis	All data are expressed as mean ± SD. Multiple comparisons were done by one-way analysis of variance (ANOVA) followed by Turkey multiple comparisons test. In the case of non-normally distributed data, the Kruskal–Wallis test was used.
Ethical Compliance	The study protocol was approved by Animal Research Committee at Tianjin University of Traditional Chinese Medicine and in accordance with the NIH Guide for the Care and Use of Laboratory Animals (2011).

24. Norcantharidin (NCTD)

Assessment Item	Specific Content
Description of Plant Material	Norcantharidin (NCTD) is a synthetic derivative of a compound used in traditional Chinese medicine that has been applied to the treatment of cancer.
Extraction Method	NCTD (Sigma-Aldrich, St. Louis, MO, USA)
Chemical Analysis	No chemical fingerprint was established.
Experimental Design	UUO model was used. NCTD was administered to mice by intraperitoneal injection (0.075 mg $kg^{-1}\cdot day^{-1}$).
Data Analysis	All data are expressed as mean ± SD. Statistical analyses were performed using SPSS v17.0 software (SPSS Inc., Chicago, IL, USA).
Ethical Compliance	All animal procedures were approved by the Animal Use and Nursing Committee of Central South University.

25. Danshen injection

Assessment Item	Specific Content
Description of Plant Material	Danshen, a traditional Chinese herbal medicine, is the dried root of the plant *Salvia miltiorrhiza* Bunge. The main active components in Danshen injection are phenolic acids, including salvianolic acid A, salvianolic acid B, danshensu, rosmarinic acid and lithospermate B.
Extraction Method	Danshen, with aqueous extracts of Radix S. miltiorrhiza as active components, was purchased from Chiatai Qingchunbao pharmaceutical.Co., Ltd. (Hangzhou, Zhejiang, China).
Chemical Analysis	The chemical fingerprint and metabolic fingerprint of Danshen for injection were used by high-performance liquid chromatography (HPLC). And chemical fingerprint was established.
Experimental Design	STZ-induced diabetic rats model was used, intraperitoneally administered with low-dose Danshen injection (0.5 ml/kg/day, L-DS) and high-dose Danshen injection (1 ml/kg/day, H-DS) for 6 weeks.
Data Analysis	All data are expressed as mean ± SEM. The results were analyzed by one-way ANOVA followed by a Bonferroni post hoc test for multiple comparisons.
Ethical Compliance	The experimental procedures were in accordance with the National Institutes of Health Guide and approved by Committee for the Care and Use of Laboratory Animals at Zhejiang University.

25. Curcumin

Assessment Item	Specific Content
Description of Plant Material	Curcumin, an active component of *Curcuma longa L.*, was first reported to have antibacterial functions in 1949.
Extraction Method	Curcumin (Sigma, USA) was suspended in 2% dimethyl sulfoxide (DMSO) with 98% saline.
Chemical Analysis	No chemical fingerprint was established.
Experimental Design	UUO model was used. The rats were gavaged with 50 mg/kg or 100 mg/kg curcumin daily for 7 days.
Data Analysis	All data are expressed as mean ± SD. Student's t-test or one-way analysis of variance (ANOVA) were conducted to calculate the statistical differences. Two-way ANOVA was used when there were two different factors.
Ethical Compliance	The Affiliated Hospital of Kunming University of Science and Technology (Kunming, China) approved all animal experiments (Permit Number: 2018GJ178).

26. Saponins from *Panax japonicus* (SPJs)

Assessment Item	Specific Content
Description of Plant Material	Saponins is a class of phytochemicals present in various plant species, including *Panax japonicus* (*P. japonicus*). And the saponins and non-saponins in ginseng have important medicinal value. The main components of SPJs include *Ginsenoside Re*, *panax japonicus* V, *panax japonicus* IV, *panax japonicus* IVa and *panax japonicus* 2.
Extraction Method	The roots of *P. japonicas* were purchased from Enshi Chunmuying Medicinal Materials Planting Base (Enshi, Hubei, China), and total saponins were extracted from the roots of *P. japonicas* with the extraction method of SPJs based on our previous research.
Chemical Analysis	No chemical fingerprint was established.
Experimental Design	Aging male Sprague Dawley (SD) rats model was used. Rats in SPJ-L group (10mg/kg/d) and SPJ-H group (60mg/kg/d) were administrated with SPJs at the indicated doses by gavage beginning from the 18th month to the 24th month.
Data Analysis	All data are expressed as mean ± SD. The significant difference in the mean value between groups was tested with one-way analysis of variance (ANOVA).
Ethical Compliance	All animal experiments were complied with the Experimental Animal Operating Standards of China Three Gorges University and were approved by the Ethics Committee of this university.

27. Honokiol (HKL)

Assessment Item	Specific Content
Description of Plant Material	An effective ingredient extracted from *Houpoea officinalis*.
Extraction Method	The roots of *P. japonicas* were purchased from Enshi Chunmuying Medicinal Materials Planting Base (Enshi, Hubei, China), and total saponins were extracted from the roots of *P. japonicas* with the extraction method of SPJs based on our previous research.
Chemical Analysis	No chemical fingerprint was established.
Experimental Design	UUO rats model was used. HKL (5 mg/kg) was administered by daily intraperitoneal injection for 7 d prior to UUO surgery and continued for 10 d after UUO surgery.
Data Analysis	All data are expressed as mean ± SD. Multiple comparisons were examined for significant differences using analysis of variance (ANOVA), followed by individual comparison with Tukey's post hoc test.
Ethical Compliance	The animal experiment protocol was reviewed and approved by the Institutional Animal Care and Use Committee of Jeonbuk National University, Jeonju, Korea (CBNU 2016-19, approved on 29 February 2016).

27. Carnosic Acid (CA)

Assessment Item	Specific Content
Description of Plant Material	CA is a naturally occurring catecholic diterpene, which is mainly found in the species of Lamiaceae family. It is highly abundant in *Rosmarinus officinalis L.* and *Salvia officinalis L.*.
Extraction Method	CA was obtained from Sigma-Aldrich Chemical Company, St. Louis, USA.
Chemical Analysis	No chemical fingerprint was established.
Experimental Design	Swiss albino mice were used. Mice were treated with CA (10 mg/kg body weight, p.o. via oral gavage, once daily) for two weeks; CA was dispersed in distilled water containing 1% tween 80.
Data Analysis	All data are expressed as mean ± SD. The statistical analysis was performed using a one-way analysis of variance (ANOVA) followed by Dunnett's t-test in the GraphPad InStat software (version 3.05), USA.
Ethical Compliance	The experiment was approved (Ref. no.: AEC/PHARM/1701/09/2017) by the institutional animal ethical committee (Reg. no.: 0367/01/C/CPCSEA, UGC, India).

28. Ginsenoside-Rg1 (G-Rg1)

Assessment Item	Specific Content
Description of Plant Material	Ginsenoside-Rg1 (G-Rg1) is an agent isolated from *Panax ginseng* that exerts anti-fibrotic effects.
Extraction Method	G-Rg1 (68317; Sigma-Aldrich, St. Louis, MO, U.S.A.)
Chemical Analysis	No chemical fingerprint was established.
Experimental Design	UUO Rat model was used. G-Rg1 was administered to the rats by intraperitoneal injection at a dose of 50 mg/kg per day based on our previous results.
Data Analysis	All data are expressed as mean ± SD. Multiple comparisons among groups were determined by a one-way ANOVA followed by Student–Newman–Keuls test.
Ethical Compliance	All animal experiments were approved by the Animal Care and Use Committee of the Kunshan First People's Hospital.

29. *Taxus chinensis*

Assessment Item	Specific Content
Description of Plant Material	*Taxus chinensis* is a valuable plant, which belongs to the Taxaceae family. There are many kinds of water soluble bioactive substances in Taxaceae plants, such as polysaccharides and flavones.
Extraction Method	*Taxus chinensis* was purchased from Shanghai Zhong-De Chinese Materia Medica Co., Ltd. (Shanghai, China). After 30 min soaking in the water, *Taxus chinensis* was boiled under water heat-reflux for 1 h twice.
Chemical Analysis	No chemical fingerprint was established.
Experimental Design	Diabetic nephropathy rat model was used. The DN rats were treated with Taxus chinensis orally (0.32, 0.64, and 1.28 g/kg) once a day for 8 weeks.
Data Analysis	All data are expressed as mean ± SD. One-way analysis of variance (ANOVA) was used to analyze the differences between groups. If any significant changes were found, post hoc comparisons were performed using Fisher's Protected Least Significant Difference (PLSD).
Ethical Compliance	Procedures involving animals and their care were approved by the Animal Ethical Committee of School of Pharmacy, Fudan University (Permit Number: 2013-3).

30. Yi Shen Pai Du Formula (YSPDF)

Assessment Item	Specific Content
Description of Plant Material	YSPDF is an innovative Chinese medicine prescribed on the basis of containing *Astragali radix* and *Rhei radix et rhizome*. It also contains two additional traditional Chinese medicines: *Hirudo* (Shui Zhi) and *Bombyx batrytocatus* (Jiang Can).
Extraction Method	YSPDF was prepared using extractions from four traditional Chinese medicines: *Astragali radix* (Huang Qi) (lot no. 20170516), *Rhei radix et rhizome* (Da Huang) (lot no.20170411), *Hirudo* (Shui Zhi) (lot no. 20170520), and *Bombyx batrytocatus* (Jiang Can) (lot no. 20170510). All the botanical and animal names are recorded and can be validated using http://mpns.kew.org/mpns-portal/?_ga=1.111763972.1427522246.1459077346. Crude YSPDF was purchased from Bozhou Jinshaotang Chinese Medicine Decoction Co., Ltd. The ratio of *Astragali radix*, *Rhei radix et rhizome*, *Hirudo*, and *Bombyx batrytocatus* in the formula was 25: 5: 3: 3. This crude YSPDF was powdered and subjected to reflux extraction with 10 volumes of water for 1.5 h. The aqueous extracts were filtered and collected. The extraction was repeated twice with the method described above, and then, all the extracts were evaporated to dryness under reduced pressure. Finally, 1 g of YSPDF extracts were equivalent to 1.44 g of original crude material. The pulverized YSPDF extracts were dispersed and dissolved in distilled water for animal experiments.
Chemical Analysis	The quality of the extracted YSPDF was analyzed using reversed phase high-performance liquid chromatography (RP-HPLC). And chemical fingerprint was established.
Experimental Design	Db/db mice were administered intragastrically with 0.4 mL of YSPDF (2 g/kg) once daily. Drug treatment lasted for 8 weeks.
Data Analysis	Differences were determined by one-way ANOVA test and Dunnett's test. All results are presented as the mean ± SEM.
Ethical Compliance	The experiment was approved by the Institutional Animal Ethics Committee (Approval No.: 2019S938).

31. Liuwei Dihuang Pil

Assessment Item	Specific Content
Description of Plant Material	Herb couple *Rehmannia glutinosa* Libosch and *Cornus officinalis* Sieb
Extraction Method	*Cornus officinalis* and *Rehmannia glutinosa* crude material were purchased from Sanyue Traditional Chinese Medicine Slices Co., Ltd (Batch No. 220422, 220617)
Chemical Analysis	The laboratory has done this before, but it is not mentioned in this paper.
Experimental Design	CKD model induced by adenine, 1 g/ml concentrated liquid prepared to meet the administration dose of 4 ml/kg. Duration of administration is 3 weeks.
Data Analysis	For comparing average numbers of members across groups, analysis of variance and Tukey's honest significance difference post hoc test were used. All results are presented as the mean ± SD.
Ethical Compliance	The experiment was approved by the Institutional Animal Ethics Committee (Approval No.: 20200826-003).

32. Shenkang VII Recipe (SK-7)

Assessment Item	Specific Content
Description of Plant Material	Shenkang VII recipe (SK-7) is a classical TCM formula consisting of *Rhizoma Atractylod* (Cangzhu), *Phellodendron bark* (HuangBo), *Smilax glabra* (Tufuling), *Rumex nepalensis Spreng* (Tudahuang), *Bolbostemma paniculatum* (Tubeimu), *Achyranthes aspera* (Tuniuxi), *Astragalus membranaceus* (Huangqi) and *Rhodiola rosea* (Hongjingtian). SK-7 formula is taken with decoction, which is commonly used in CKD patients.
Extraction Method	SK-7 was obtained from Hubei Tianji Traditional Chinese Medicine Pieces Co., Ltd (China). According to the original prescription from the clinical dosage, the daily preliminary proportion of the 8 herbs were Cang Zhu (10 g), Huangbo (10 g), Tufuling (30 g), Tudahuang (20 g), Tubeimu (10 g), Tuniuxi (10 g), Huangqi (30 g) and Hongjingtian (20 g). To obtain the aqueous extract of SK-7, all medical materials were soaked in 800 mL distilled water for 30 min, and then decocted by boiling in distilled water twice, 1 h per time. The solution was then dried under microwavedrier to obtain SK-7 powder.
Chemical Analysis	No chemical fingerprint was established.
Experimental Design	UUO model plus low to high doses of SK-7 (0.5, 1.0, or 2.0 g/kg/day, for 14 days).
Data Analysis	All data are expressed as mean ± SD. Analysis of variance (ANOVA) was used to analyze intergroup comparisons and multiple groups.
Ethical Compliance	All the animal experiments were conducted in accordance with the Animal Care and Use Committee of the Institute of Materia Medica, China.

33. Kangxianling decoction

Assessment Item	Specific Content
Description of Plant Material	Kangxianling decoction was widely used in patients with chronic kidney disease, which could improve symptoms such as poor appetite, edema, and fatigue. The gradient of the kangxianling decoction consisted of 15 g of radix salvia miltiorrhizae (Dan Shen), 9 g of prepared rhubarb (Zhi Da Huang), 12 g of peach kernel (Tao Ren), 12 g of radix angelicae ainensis (Dang Gui),15 g of badix achyranthis bidentatae (Niu Xi).
Extraction Method	All crudes of kangxianling decoction drugs were purchased from Shanghai Kang Qiao Chinese Herbs Co., LTD.
Chemical Analysis	No chemical fingerprint was established.
Experimental Design	5/6 N induced renal fibrosis model was used, kangxianling decoction (21 g/kg/d) administered by gavage for four months.
Data Analysis	All data are expressed as mean ± SD. Data were analyzed using one-way analysis of variance (ANOVA) test for comparison of multiple independent sample group.
Ethical Compliance	All animals were handled in accordance with the protocol approved by the Ethics Committee of Animal Research at the college of Shanghai University of Traditional Chinese Medicine.

34. Qingxuan Jiangya Decoction (QXJYD)

Assessment Item	Specific Content
Description of Plant Material	QXJYD has long been used in China for the clinical treatment of hypertension. Many active components have been identified in QXJYD, such as rhynchophylline, gastrodin, and baicalin, which have been shown to possess antihypertensive activities.
Extraction Method	All crudes of kangxianling decoction drugs were purchased from Shanghai Kang Qiao Chinese Herbs Co., LTD.
Chemical Analysis	HPLC was used to determine the content, but no chemical fingerprint was established.
Experimental Design	Rats in QXJYD group were given oral treatment of 60 mg/kg/day QXJYD.
Data Analysis	All data are expressed as mean ± SD. One-way analysis of variance (ANOVA) was used to determine statistical significance.
Ethical Compliance	All animal experiments were performed strictly in accordance with international ethical guidelines and the National Institutes of Health Guide concerning the care and use of laboratory animals and were approved by the Institutional Animal Care and Use Committee of Fujian University of Traditional Chinese Medicine.

35. Shenqi detoxification granule (SDG)

Assessment Item	Specific Content
Description of Plant Material	SDG, a traditional Chinese herbal formula, has been used in the clinic for the treatment of CKD for many years. SDG contains 12 herbs including *Astragalus membranaceus* (Fisch.) Bge, *Salvia miltiorrhiza* Bge, *Angelica sinensis* (Oliv.) Diels, *Rheum officinale* Baill., *Euryale ferox* Salisb., *Eclipta prostrata* L., *Semiaquilegia adoxoides* (DC.) Makino, *Imperata cylindrica Beauv. var. major* (Nees) C. E. Hubb., *Aconitum carmichaelii* Debx., *Epimedium brevicornu* Maxim, *Rosa laevigata* Michx., *Hedyotis diffusa* Willd.
Extraction Method	These twelve ingredients were mixed in proportion, boiled with distilled water twice, and the resulting decoction was mixed and filtered using filter paper. Then, the filtered solution was collected and concentrated to a relative density. Finally, clear paste and excipient were mixed to make sugar-free granules, then dried, granulated, packed (10 g/bag), and labeled, which contained 20 g granules of 217 g crude drugs. Then the granules were made into 0.4 mg/mL liquid and kept at 4°C.
Chemical Analysis	Chemical fingerprint was established in their previous study.
Experimental Design	UUO model was used. UUO + SDG group were gavaged with 2 mL SDG daily for 4 weeks and injected with 0.5 mL normal saline through tail vein each week for 4 weeks.
Data Analysis	All data are expressed as mean ± SD. The statistical significance of differences were calculated using the t-test and one-way analysis of variance (ANOVA).
Ethical Compliance	All experimental procedures were approved by the Institutional Animal Care and Use Committee of Shandong Provincial Hospital Affiliated to Shandong University (No. 2017-208).

36. Modified Huangqi Chifeng decoction (MHCD)

Assessment Item	Specific Content
Description of Plant Material	MHCD is an empirical formulation by Prof. Yu Zhang, a Registered Chinese Medicine Practitioner and Chinese medicine nephrologist working at Xiyuan Hospital, China Academy of Chinese Medical Sciences. MHCD contains seven Chinese herbal medicines including Radix Astragali (the root of *Astragalus mongholicus* Bunge), Radix Paeoniae Rubra (the root of *Paeonia lactiflora* Pall.), Radix Saposhnikoviae [the root of *Saposhnikovia divaricata* (Turcz. ex Ledeb.) Schischk.], Semen Euryales (the dried kernel of the ripe seed of *Euryale ferox* Salisb.), Fructus Rosae Laevigatae (the fruit of *Rosa laevigata* Michx.), Rhizoma Dioscoreae Nipponicae (the rhizome of *Dioscorea nipponica* Makino), and Hedyotis diffusa (whole herb of *Hedyotis diffusa* Willd.).
Extraction Method	The MHCD comprises the following ingredients: 30 g of Radix Astragali, 10 g of Radix Paeoniae Rubra, 10 g of Radix Saposhnikoviae, 20 g of Semen Euryales, 10 g of Fructus Rosae Laevigatae, 20 g of Rhizoma Dioscoreae Nipponicae, and 20 g of Hedyotis diffusa. These ingredients were used for preparing a water decoction by the Chinese Medicine Preparation Research Laboratory at Xiyuan Hospital of the China Academy of Chinese Medical Sciences.
Chemical Analysis	UPLC-MS/MS analysis was conducted to identify the main chemical components of the decoction, and a chemical fingerprint was established.
Experimental Design	An IgA nephropathy rat model was used. Low-dose MHCD (6.25 g/kg), medium-dose MHCD (12.50 g/kg), and high-dose MHCD (25.00 g/kg) groups, intragastric drug administration occurred for 8 weeks.
Data Analysis	All data are expressed as mean ± SD. The differences between groups were compared using one-way analysis of variance (ANOVA).
Ethical Compliance	The experiment was approved by the Institutional Animal Ethics Committee (Approval No.: J20180016).

37. Xiexin Decoction (XXD)

Assessment Item	Specific Content
Description of Plant Material	Xiexin decoction, a herbal therapeutic agent commonly used in traditional Chinese medicine, is recognized for its beneficial effects on diabetic nephropathy exerted through the combined action of multiple components, including Rhizoma Coptidis alkaloids (A), Radix et Rhizoma Rhei polysaccharides (P), and Radix Scutellaria flavones (F). It has been used in the treatment of diabetes for 1300 years.
Extraction Method	APF was formulated in a 1:2:1 (A:P:F) ratio, with the preparation and quality control methods of these herbal components performed as previously described.
Chemical Analysis	UPLC analysis was conducted to identify the main chemical components of the decoction, but no chemical fingerprint was established.
Experimental Design	An db/db diabetic model was used. Low dose of APF (APF 300 mg/kg, db/db mice receiving 300 mg/kg APF, containing 75, 150, and 75 mg/kg of A, P, and F, respectively); high dose of APF (APF 600 mg/kg, db/db mice receiving 600 mg/kg of APF, containing 150, 300, and 150 mg/kg of A, P, and F, respectively). All treatments were administered by intragastric gavage once a day, for 8 weeks.
Data Analysis	All data are expressed as mean ± SD. Statistical differences between the groups were evaluated by one-way analysis of variance (ANOVA). The dose-dependent nature of the relationship was evaluated by linear correlation analysis.
Ethical Compliance	All animal experiments were performed with the approval of the Institutional Animal Care and Use Committee of Shanghai University of Traditional Chinese Medicine (Approval Number: 2012012).

38. Jieduquyuzishen Prescription (JP)

Assessment Item	Specific Content
Description of Plant Material	JP is composed of ten herbs including *Artemisia annua L.*, *Cimicifuga heracleifolia Kom.*, *Hedyotis diffusa Willd*, *Paeonia suffruticosa Andr.*, *Trionyx sinensis Wiegmann*, *Centella asiatica* (L.) Urb., *Citrus medica* L. *var. sarcodactylis Swingle*, *Glycyrrhiza uralensis Fisch*, *Paeonia lactiflora Pall.*, and *Rehmannia glutinosa Libosch*. It has been widely used in the treatment of lupus nephritis in China and has achieved good clinical effects.
Extraction Method	Corresponding to the common dose for adult humans, which was obtained from Zhejiang Chinese Medical University Medical Pieces., LTD (Hangzhou, China). After soaking in water (w/v, 1/10) for 1 h, the mixed herbs were boiled for 2 h for extraction. The residue was extracted again for another 2 h. The filtrates were collected, combined, and concentrated to 1.56 g crude drug/mL and then preserved at 4°C and rewarmed before administration.
Chemical Analysis	No chemical fingerprint was established.
Experimental Design	MRL/lpr mice model was used. Mice of the JP group were administered JP (18 ml/kg body weight (bw) per day, i.g.).
Data Analysis	All data are expressed as mean ± SD. The statistical significance was determined by Student's t-test and one-way ANOVA analysis.
Ethical Compliance	All the above animal studies were approved by the Animal Experiment Ethics Committee of Zhejiang Chinese Medical University.
